# Synthesis, Structure, DNA Interaction, and SOD Activity of Three Nickel(II) Complexes Containing L-Phenylalanine Schiff Base and 1,10-Phenanthroline

**DOI:** 10.1155/2018/8478152

**Published:** 2018-07-05

**Authors:** Peiran Zhao, Shanshan Zhai, Jianfang Dong, Lei Gao, Xinru Liu, Lei Wang, Jinming Kong, Lianzhi Li

**Affiliations:** ^1^School of Chemistry and Chemical Engineering, Liaocheng University, Liaocheng 252059, China; ^2^Zhong Yuan Academy of Biological Medicine, Liaocheng People's Hospital, Liaocheng 252000, China; ^3^School of Environmental and Biological Engineering, Nanjing University of Science and Technology, 200 Xiaolingwei, Nanjing 210094, China

## Abstract

Three hexacoordinated octahedral nickel(II) complexes, [Ni(sal-L-phe)(phen)(CH_3_OH)]·CH_3_OH (**1**), [Ni(naph-L-phe)(phen)(CH_3_OH)] (**2**), and [Ni(*o*-van-L-phe)(phen)(CH_3_OH)]·5CH_3_OH (**3**) (sal-L-phe = a Schiff base derived from salicylaldehyde and L-phenylalanine, naph-L-phe = a Schiff base derived from 2-hydroxy-1-naphthaldehyde and L-phenylalanine, *o*-van-L-phe = a Schiff base derived from *o*-vanillin and L-phenylalanine, and phen = 1,10-phenanthroline), have been synthesized and characterized by elemental analysis, IR spectra, and single-crystal X-ray diffraction. The interactions of these complexes with CT-DNA were studied by UV-Vis absorption spectroscopy, fluorescence spectroscopy, circular dichroism spectroscopy, and viscosity measurements. The binding constant (*K*_b_) values of 1.82 × 10^4^ M^−1^ for **1**, 1.96 × 10^4^ M^−1^ for **2**, and 2.02 × 10^4^ M^−1^ for **3** suggest that each of these complexes could bind with DNA in a moderate intercalative mode. Complex **3** exhibited a stronger interaction with CT-DNA than complexes **1** and **2**. In addition, the superoxide scavenging activity of these complexes was investigated by the nitrotetrazolium blue chloride (NBT) light reduction method, and the results showed that they exhibited a significant superoxide scavenging activity with the IC_50_ values of 4.4 × 10^−5^ M for complex **1**, 5.6 × 10^−5^ M for complex **2**, and 3.1 × 10^−5^ M for complex **3**, respectively.

## 1. Introduction

Bioinorganic chemistry usually studies the interaction of inorganic elements with the organism at the molecular level [1]. The interaction between small molecules and biological macromolecules has become an important research topic in bioinorganic chemistry; especially the interaction between transition metal complexes and DNA has aroused the widespread interest [2, 3]. This helps us not only to understand the life processes at the molecular level but also to promote the development of chemistry discipline itself.

Amino acid Schiff bases are usually composed of amino acids with different aldehyde or ketone carbonyl groups. Schiff base is a multidentate ligand, which plays an important role in medicinal and pharmaceutical areas [4, 5]. In recent years, studies on the amino acid Schiff bases and their metal complexes are very active. Synthesis, characterization, structure, and thermodynamic and kinetic properties of this kind of compounds have been reported, and their antibacterial, anti-inflammatory, and anticancer activities have been widely studied [6]. Therefore, it is an important research topic to study the relationship among structure, property, and biological activity of these amino acid Schiff base complexes [7].

Phenylalanine is an essential amino acid for human and other organisms. Amino acid with a side chain aromatic ring such as phenylalanine contributes mainly to the stabilization of proteins through hydrophobic interactions and the formation of hydrophilic environments [8]. In addition, it is a precursor of many aromatic compounds and of alkaloids such as curare and morphine.

Nickel is an essential trace element for the human body, and the metalloenzymes containing nickel(II) play important physiological functions in the organisms [9]. Nickel(II) complexes have been reported to act as anticonvulsant and antiepileptic agents or vitamins; they have also presented antibacterial, antifungal, antimicrobial, antioxidant, and antiproliferative/anticancer activities [10–20]. Therefore, the research of nickel(II) complexes has attracted more and more attention and become more and more important in the field of bioinorganic and coordination chemistry [21]. Recently, studies on amino acid Schiff base ligands and nickel(II) complexes have been included [22, 23].

Deoxyribonucleic acid (DNA) is an important biomolecule. The biological information stored in the DNA is expressed by means of replication, transcription, and translation. This information has the ability to guide cell growth, metabolism, and mutation. Many small molecules exert their anticancer activities by binding with DNA, thereby altering DNA replication, blocking the division of cancer cells, and resulting in the cell death [24]. The interactions of nickel(II) complexes with DNA/BSA have been reported previously [25–28]. For example, nickel(II) complexes of benzoic acid (2-hydroxy-benzylidine)-hydrazide ligands bind to DNA base pairs via intercalation and *π*-*π* stacking interactions [29]. Nickel(II) complexes containing *N*-substituted heterocyclic thiosemicarbazones have been found to exhibit remarkable DNA/protein binding and antioxidant activities [30, 31]. In addition, nickel(II) complexes have attracted the attention of researchers because of their superoxide scavenging activities and broad biological activities [32, 33].

In view of the above considerations, we have synthesized three new nickel(II) complexes of Schiff base derived from the reactions of phenylalanine and aromatic aldehydes. These complexes have been characterized by elemental analysis, infrared (IR) spectra, and single-crystal X-ray diffraction. Furthermore, the interactions of these complexes with calf thymus DNA (CT-DNA) and their superoxide scavenging activities have been investigated by using spectroscopies.

## 2. Experimental

### 2.1. Materials and Physical Measurements

L-phenylalanine was obtained from Beijing Jingke Hongda Biotechnology Co., Ltd. Salicylaldehyde, *o*-vanillin, and 2-hydroxy-1-naphthaldehyde were purchased from Alfa Aesar. CT-DNA was obtained from Beijing Biodee Biotechnology Co., Ltd. Ethidium bromide (EB) was gained from Sigma. Tris(hydroxymethyl)aminomethane (Tris), nickel(II) acetate, 1,10-phenanthroline, potassium hydroxide, and anhydrous methanol were commercially available analytical reagents.

Tris-HCl (10 mM) (10 mM NaCl, pH = 7.1) buffer solution was prepared with doubly distilled water. A solution of CT-DNA in the buffer gave a ratio of absorbance at 260 and 280 nm (*A*_260_/*A*_280_) of 1.8-1.9, reflecting that the DNA was sufficiently free of protein [34, 35].

IR spectra were recorded as KBr pellets on a Nicolet 5700 FT-IR instrument in the frequency range of 400–4000 cm^−1^. Both electronic and fluorescence spectra of the complexes were recorded using a Shimadzu UV-2550 spectrophotometer and a PerkinElmer LS55 spectrofluorometer, respectively. Circular dichroism (CD) spectra were obtained on a Jasco J-810 spectropolarimeter.

### 2.2. Synthesis of the Complexes

#### 2.2.1. Synthesis of the Complex [Ni(sal-L-phe)(phen)(CH_3_OH)]·CH_3_OH (**1**)

L-phenylalanine (0.1652 g, 1 mmol) and potassium hydroxide (0.056 g, 1 mmol) were dissolved in methanol (20 mL) at 323 K and added to a methanol solution (5.0 mL) of salicylaldehyde (0.11 ml, 1 mmol) and stirred for 1 h. Then, the solution (2.0 mL) of nickel(II) acetate tetrahydrate (0.25 g, 1 mmol) was added dropwise and stirred sequentially for 2 h. In the end, a methanol solution (4.0 mL) of 1,10-phenanthroline (0.180 g, 1 mmol) was added and continuously stirred for 3 h. The resulting solution was filtered, and then, the filtrate was placed at room temperature for two weeks; the green block single crystals suitable for X-ray diffraction analysis were gained. Anal. calc. (%) for C_30_H_29_N_3_O_5_Ni (Mr = 570.27): C, 63.19; H, 5.13; and N, 7.37%. Found: C, 63.31; H, 5.20; and N, 7.26%. IR (KBr, 4000–400 cm^−1^): 3384.9 (s, *v*_O–H_), 1636.0 (s, *v*_C=N_), 1584.7 (m, *v*_COO_^−^), 1384.7(m, *v*_COO_), 847.1 (w, *v*_Ni–O_), and 739.0 (s, *v*_Ni–N_). The mass spectrum and ^1^H NMR of the Schiff base **1**, H_2_(sal-L-phe), are shown in Figures S1 and S4, respectively.

#### 2.2.2. Synthesis of the Complex [Ni(naph-L-phe)(phen)(CH_3_OH)](**2**)

The solution of L-phenylalanine (0.1652 g, 1 mmol), potassium hydroxide (0.056 g, 1 mmol), and 2-hydroxy-1-naphthaldehyde (0.1722 g, 1 mmol) in anhydrous methanol (20 mL) was heated at 323 K for 1 h. Then, a solution (2.0 mL) of nickel(II) acetate tetrahydrate (0.25 g, 1 mmol) was added dropwise and stirred continuously for 2 h. Finally, a methanol solution (5.0 mL) of 1,10-phenanthroline (0.20 g, 1.0 mmol) was added and continuously stirred for 3 h. The resulting solution was filtered, and the brown crystals suitable for X-ray diffraction analysis were obtained from the filtrate kept at normal atmospheric temperature for two weeks. Anal. calc. (%) for C_33_H_27_N_3_O_4_Ni (Mr = 588.28): C, 67.38; H, 4.63; and N, 7.14%. Found: C, 67.29; H, 4.71; and N, 7.08%. IR (KBr, 4000–400 cm^−1^): 3423.4 (s, *v*_O–H_), 1618.8 (s, *v*_C=N_), 1537.1 (m, *v*_COO_−), 1313.0 (m, *v*_COO_−), 852.9 (w, *v*_Ni–O_), and 728.2 (s, *v*_Ni–N_). The mass spectrum and ^1^H NMR of the Schiff base **2**, H_2_(naph-L-phe), are shown in Figures S2 and S5, respectively.

#### 2.2.3. Synthesis of the Complex [Ni(*o-*van-L-phe)(phen)(CH_3_OH)]*·*5CH_3_OH (**3**)

**Scheme 1:**
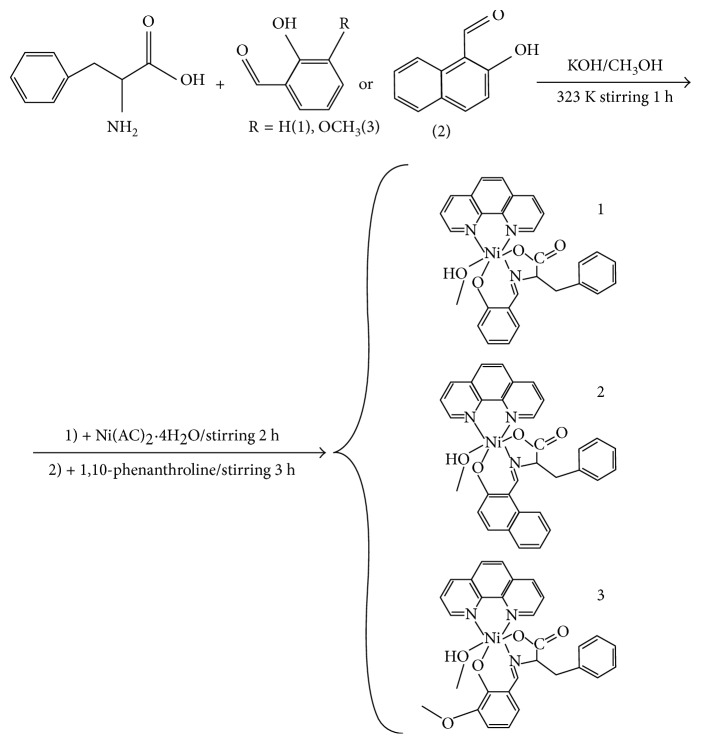
Synthetic routes for the preparation of complexes **1**, **2**, and **3**.

Synthetic routes for the preparation of complexes **1**, **2** and **3** are shown in Scheme 1. Complex **3** was prepared following the same procedure as for complex **2** except that an anhydrous methanol solution (2 mL) of *o*-vanillin (0.1522 g, 1 mmol) was used instead of 2-hydroxy-1-naphthaldehyde. The reaction solution was filtered, and the filtrate was kept at normal atmospheric temperature for two weeks; then, the light green crystals suitable for X-ray diffraction analysis were obtained. Anal. calc. (%) for C_95_H_101_N_9_O_20_Ni_3_ (Mr = 1864.98): C, 61.18; H, 5.46; and N, 6.76%. Found: C, 61.02; H, 5.50; and N, 6.64%. IR (KBr, 4000–400 cm^−1^): 3378.5 (s, *v*_O–H_), 1632.3 (s, *v*_C=N_), 1440.8 (m, *v*_COO_−), 1082.9 (m, *v*_COO_−), 855.1 (w, *v*_Ni–O_), and 545.5 (s, *v*_Ni–N_). The mass spectrum and ^1^H NMR of the Schiff base **3**, H_2_(*o*-van-L-phe), are shown in Figures S3 and S6, respectively.

### 2.3. X-Ray Crystallographic Analysis

X-ray single-crystal diffraction measurements for these nickel(II) complexes were recorded on a Bruker Smart 1000 CCD area-detecting diffractometer. Diffraction intensities for the nickel(II) complexes were collected using graphite-monochromated Mo K*α* radiation (*λ* = 0.071073 nm) at 298(2) K with the *ω*-2*θ* scan technique. The correction of semiempirical absorption data was performed using SADABS. The structures of these complexes were solved by direct methods using SHELXS-97 and subsequent Fourier difference techniques and refined anisotropically by full-matrix least-squares on *F* ^2^ using SHELXL-97 [36]. The H atoms were assigned with common isotropic displacement factors. All nonhydrogen atoms were refined with anisotropic thermal parameters. Further crystallographic data, experimental details, and refinement results for structural analyses of complexes are listed in Table 1.

Crystallographic data for the structural analysis of these nickel(II) complexes have been deposited in the Cambridge Crystallographic Data Center (CCDC no. 929664 (**1**), no. 1826530 (**2**), and no. 933859 (**3**)). Copies of the information may be obtained free of charge from http://www.ccdc.cam.ac.uk/conts/retrieving.html or from the CCDC, 12 Union Road, Cambridge, CB2 1EZ, UK (email: deposit@ccdc.cam.ac.uk).

### 2.4. DNA Binding Experiment

The binding experiments of these complexes with CT-DNA were conducted in 10 mM Tris-HCl/10 mM NaCl (pH = 7.1) buffer solution. The determination of UV-Vis absorption spectra was carried out by adding the increasing amounts of DNA (from 0 to 9.0 × 10^−5^ M) to each of these complexes with a fixed concentration of 1.5 × 10^−5^ M. The spectra were measured in the wavelength range of 200−500 nm.

For the fluorescence spectra, we have prepared the different concentrations of the samples with Tris-HCl buffer solution via keeping constant concentration of the EB-DNA system (*c*_EB_ = 10 *μ*M and *c*_DNA_ = 10 *μ*M) and increasing the concentration of complexes **1**, **2**, and **3**, respectively. The mixed solutions were allowed to stand for 30 min to equilibrium, and the fluorescence emission spectra were recorded from 530 to 700 nm at an excitation wavelength of 510 nm. Each spectrum was measured at a scan speed of 300 nm·min^−1^, and the widths of both the excitation and emission slits were set to 10.0 nm.

For CD spectra, a series of samples were prepared by adding the increasing concentrations of these complexes to constant concentration of DNA (10.0 mM) in Tris-HCl buffer solution. All the sample solutions were scanned in the range of 220–320 nm with a scan speed of 200 nm·min^−1^. Each determination of the CD spectrum was the average of three scans with 1.0 nm path resolution and 1 s response time. The optical chamber of the CD spectrometer was deoxygenated with dry nitrogen before use and kept in a nitrogen atmosphere during experiments. The final spectra were background-corrected by subtracting the corresponding buffer spectra.

Viscosity experiments were carried out on an Ubbelodhe viscometer at a constant temperature of 30°C in a thermostated bath. A series of samples were prepared using Tris-HCl buffer solution. The concentration of CT-DNA (10.0 mM) was kept constant, while the ratios *r* of [*c*(complex)/*c*(DNA)] were 0, 0.02, 0.04, 0.06, 0.08, and 0.10, respectively. Each sample was incubated in water bath for 30 min, and the flow times of the solutions through the capillary were repeatedly measured three times using a digital stopwatch. The DNA viscosity was calculated according to *η* = (*t* − *t*_0_)/*t*_0_, where *t*_0_ is the time required for the buffer solution to flow through the capillary. The data were calculated as (*η*/*η*_0_)^1/3^ versus *r* (*r* = *c*_(complex)_/*c*_(DNA)_), where *η*_0_ is the relative viscosity of the DNA solution without complex and *η* is the viscosity of DNA in the presence of complexes.

### 2.5. SOD Activity Assay

The superoxide dismutase (SOD) activities of these complexes were determined by measuring their scavenging degree for the superoxide anion radical. In the absence and presence of different concentrations of these complexes, the standard test solutions containing 6.2 *μ*M riboflavin, 83.0 mM *N*,*N*,*N*′,*N*′-tetramethylethylenediamine (TMEDA), and 10.0 mM NBT in 10.0 mM phosphate buffer (pH = 7.8) were irradiated with constant-intensity cold light at room temperature. The absorbances at 560 nm were recorded by using a UV-Vis absorption spectrophotometer in an interval of 1 min for 10 min.

## 3. Results and Discussion

### 3.1. IR Spectra of the Complexes

**Figure 1:**
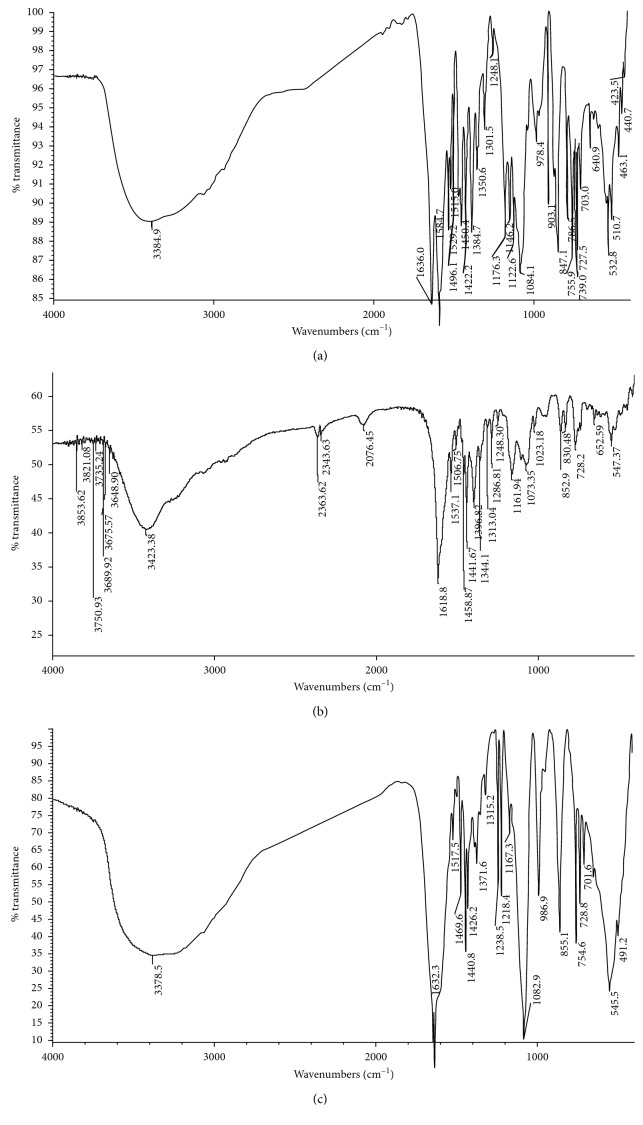
FTIR spectra of the title complexes **1** (a), **2** (b), and **3** (c).

In IR spectra of complexes **1**, **2**, and **3** (Figure 1), the broad and strong absorption at 3384.9 cm^−1^ for **1**, 3423.4 cm^−1^ for **2**, and 3378.5 cm^−1^ for **3** is due to the O–H stretching vibration. The very sharp single absorption at 1636.0 cm^−1^ for **1**, 1618.8 cm^−1^ for **2**, and 1632.3 cm^−1^ for **3** can be assigned to the imine group (C = N) stretching frequency of the coordinated Schiff base ligand. Two moderate absorptions at 1584.7 cm^−1^ and 1384.7 cm^−1^ for complex **1**, 1537.1 cm^−1^ and 1313.0 cm^−1^ for complex **2**, and 1440.8 cm^−1^ and 1082.9 cm^−1^ for complex **3** are attributed to asymmetric and symmetric stretching vibrations of the COO^–^ group, respectively [37]. The frequency separation (Δ*ν* = *ν*(COO^–^)_asy_ – *ν*(COO^–^)_sym_) is greater than 200 cm^−1^, which suggests that the carboxylate is unidentate [38]. Furthermore, the absorption bands at 847.1 cm^−1^ and 739.0 cm^−1^ for **1**, 852.9 cm^−1^ and 728.2 cm^−1^ for **2**, and 855.1 cm^−1^ and 545.5 cm^−1^ for **3** may reasonably be assigned to Ni–O and Ni–N vibrations, respectively.

### 3.2. The Structure of the Nickel(II) Complexes **1**, **2**, and **3**

**Figure 2:**
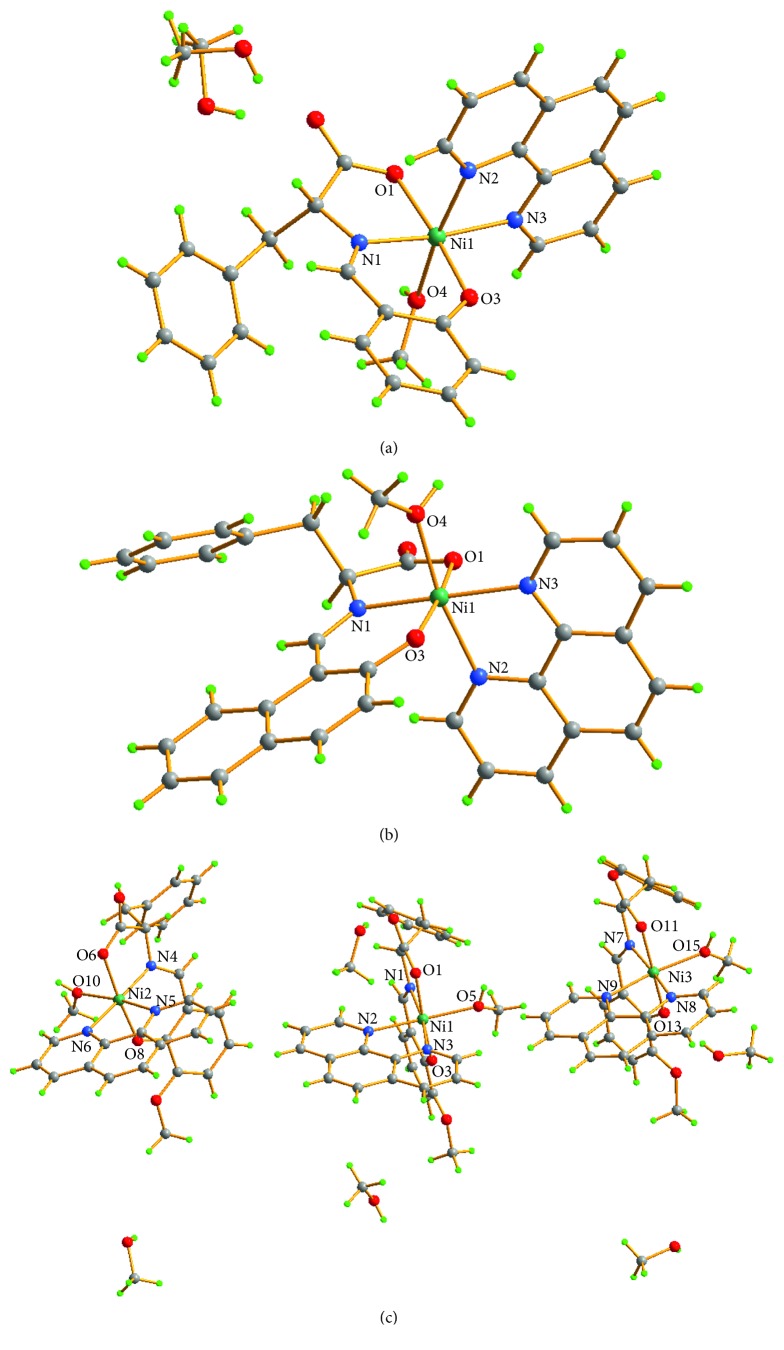
Molecular structures of complexes **1** (a), **2** (b), and **3** (c).

Figure 2 is the molecular structure of complexes **1**, **2**, and **3**. X-ray single-crystal diffraction analysis showed that complex **1** belongs to the monoclinic system, C2/c space group, and both complexes **2** and **3** belong to the triclinic system, P-1 space group. In each asymmetric structure unit of complex **1**, the equatorial coordination to Ni(II) atom is provided by N3 atom from the phenanthroline ligand and O1, N1, and O3 atoms from the Schiff base ligand. The two axial sites of the octahedron are occupied by N2 atom from the phenanthroline ligand and O4 atom from the methanol ligand, with the trans angle of N2–Ni–O4 = 170.75(11)°. The distance from Ni1 atom to the equatorial plane is 0.0753(14) Å, and the distances from O4 and N2 to the equatorial plane are 2.117(3) Å and 2.116(3) Å, respectively. The plane of the 1,10-phenanthroline ligand is almost perpendicular to the octahedral equatorial plane where Ni(1) lies, and the angle is 87.234(54)°. The Schiff base ligand forms a five-membered ring of Ni1–O1–C1–C2–N1 and a six-membered ring of Ni1–N1–C10–C11–C12–O3 surrounding the Ni1 atom, in which the dihedral angle formed by the plane of the two rings is 3.821(80)°. It is shown that these two rings are not really coplanar, but the stability of the complex is increased.

In complex **2**, O4 atom from the methanol ligand and N2 atom from the phenanthroline ligand lie in the axial positions of the octahedral geometry with the trans angle of 170.29(12)°. And N3 atom from the phenanthroline ligand and O1, N1, and O3 atoms from the Schiff base ligand are located in the equatorial plane of the octahedron. The Schiff base ligand coordinates to Ni1 atom to form one five-membered ring and one six-membered ring, thus increasing the stability of the complex.

There are three structurally identical complex molecules in the structural unit of complex **3**, in which each individual molecule has a Ni atom coordinated with a tridentate Schiff base ligand, a bidentate phenanthroline ligand, and a methanol molecule. In each individual complex molecule, the axial position of the octahedron is occupied by the O atom on the methanol ligand and the N atom on the phenanthroline ligand. The three atoms in the Schiff base and a N atom of phenanthroline occupy its octahedral equatorial plane position, respectively. For example, the equatorial coordination to Ni1 is provided by N3 atom from the phenanthroline ligand and O1, N1, and O3 atoms from the Schiff base ligand, while N2 atom from the phenanthroline ligand and O5 atom from the methanol ligand are located in two axial sites of the octahedron with the trans angle of N2–Ni1–O5 = 169.7(2)°. The Schiff base ligand forms a five-membered ring and a six-membered ring surrounding the Ni(II) atom, and the dihedral angles formed by the planes of the two rings are 3.970(317)°, 9.895(283)°, and 12.639(434)°, respectively, in each individual molecule, which increases the stability of the complex.

**Figure 3:**
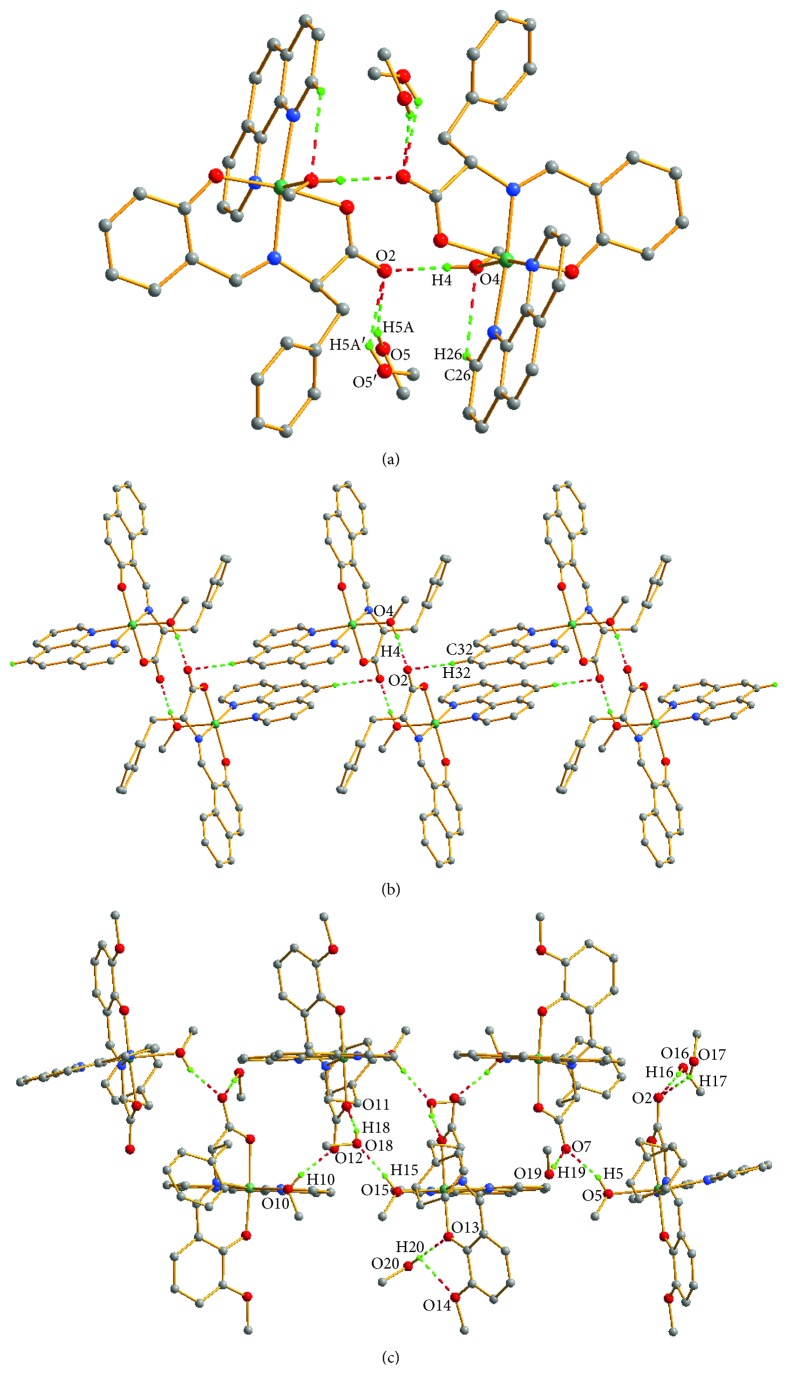
2D supramolecular network in complexes **1** (a), **2** (b), and **3** (c) formed by intermolecular hydrogen-bonding interactions. Some H atoms and C atoms were omitted for clarity.

In view of all these observations, each asymmetric structural unit in complexes **1**, **2**, and **3** has formed a six-coordination environment with a distorted octahedral structure. The selected bond lengths and angles around the metal center are given in Table 2. There are some intermolecular C–H⋯O and O–H⋯O hydrogen bonds linking the molecules to form a dimer or 2D structures as shown in Figure 3. In the crystal of complex **1**, the unordered methanol molecule is linked to the main molecule by O5′–H5A′⋯O2 and O5–H5A⋯O2 intrahydrogen bonds. And two main molecules in the crystal form a dimer by an O4–H4⋯O2 intermolecular hydrogen bond. In the crystal of complex **2**, a one-dimensional structure is formed through the intermolecular hydrogen bonds O4–H4⋯O2 and C32–H32⋯O2. In the crystal of complex **3**, five methanol molecules connect with three main molecules through O16–H16⋯O2, O17–H17⋯O2, O19–H19⋯O7, O18–H18⋯O11, O20–H20⋯O13, and O20–H20⋯O14 intermolecular hydrogen bonds, together with O15–H15⋯O18, O5–H5⋯O7, and O10–H10⋯O12 to form a two-dimensional structure. Hydrogen bond lengths and bond angles for the complexes are shown in table 3.

### 3.3. DNA Binding Studies

#### 3.3.1. UV-Vis Absorption Spectra

**Figure 4:**
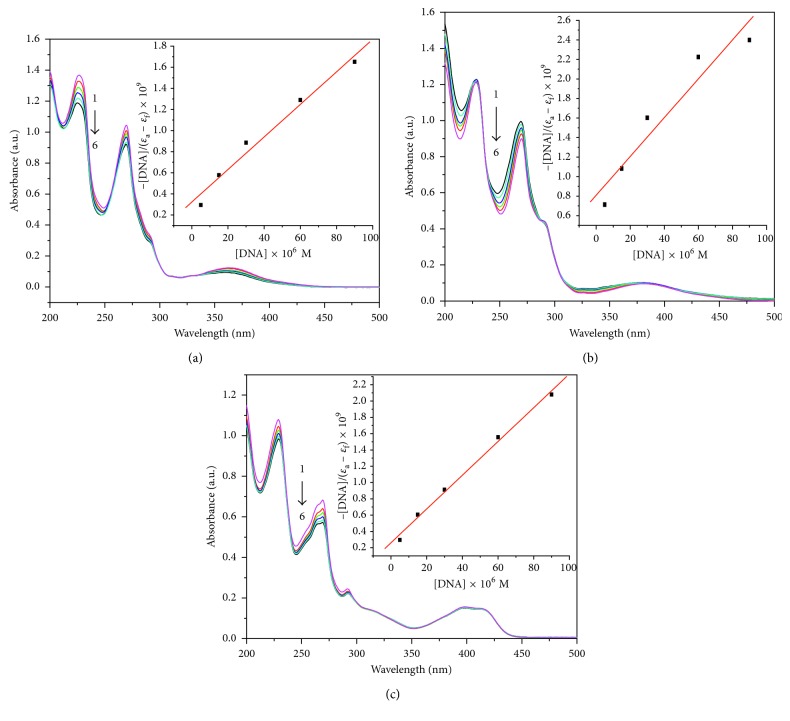
UV-Vis spectra of complexes **1** (a), **2** (b), and **3** (c) in the absence and presence of CT-DNA; the complex concentration was 1.5 × 10^−5^ M, and DNA concentrations were 0, 0.5 × 10^−5^, 1.5 × 10^−5^, 3.0 × 10^−5^, 6.0 × 10^−5^, and 9.0 × 10^−5^ M corresponding to the curves from 1 to 6, respectively. The arrow shows the intensity change on increasing the DNA concentration.

Electronic absorption spectroscopy is one of the commonly used methods for studying the interaction of small molecules with DNA. In the presence of DNA, the microenvironment of the ligand of the complex is affected, thus resulting in changes in the absorption intensity and wavelength of the complex. In general, UV-Vis absorption spectrum does not change appreciably when the metal complex interacts with DNA by electrostatic or trench interaction. But the absorption peak of the complex occurs red-shifted and the absorption intensity decreases when the metal complex interacts with DNA by intercalation [39]. This phenomenon can be explained by the generation of *π*-electron stacking action due to the ligand inserted into the DNA base, in which the *π* ^*∗*^ orbital within the ligand is coupled to the *π* orbital of the base, resulting in a reduction of the transition energy of *π* → *π* ^*∗*^ and generation of the red shift phenomenon. UV absorption spectra of these complexes with different concentrations of CT-DNA are shown in Figure 4. The absorbances at 268 nm of these complexes had an obvious hypochromic effect and a slightly red shift, showing a binding property of the three complexes with DNA, which also indicates that these complexes interact with CT-DNA mainly through the intercalative mode [40, 41].

In order to measure the strength of DNA binding with the complex quantitatively, the intrinsic binding constant (*K*_b_) of the complex with CT-DNA can be calculated by using the following equation [42]:

(1) $$\begin{array}{c} \frac{\left[\mathrm{DNA}\right]}{\varepsilon_{a}-\varepsilon_{f}}=\frac{\left[\mathrm{DNA}\right]}{\varepsilon_{b}-\varepsilon_{f}}+\frac{1}{K_{b}\left(\varepsilon_{b}-\varepsilon_{f}\right)}, \end{array}$$

where [DNA] is the concentration of DNA in base pairs, *ε*_a_ is the molar extinction coefficient of the complex in the presence of DNA, and *ε*_f_ and *ε*_b_ are the molar extinction coefficients for the free complex and the fully DNA-bound complex, respectively. *K*_b_ can be obtained from the ratio of the slope to the intercept in the plot of [DNA]/*ε*_a_ − *ε*_f_ versus [DNA]. In this experiment, the calculated intrinsic binding constant *K*_b_ values were 1.82 × 10^4^, 1.96 × 10^4^, and 2.02 × 10^4^ M^−1^ for complexes **1**, **2**, and **3**, respectively. These values suggest that the binding strength of all the three nickel(II) complexes to DNA is weaker than that of ethidium bromide (EB) to DNA (*K*_b_ = 3.3 × 10^5^ L·mol^−1^) [43], indicating that these complexes have a weaker binding ability to DNA, which may be due to the deformed octahedral structure of these complexes.

#### 3.3.2. Fluorescence Spectroscopy

**Figure 5:**
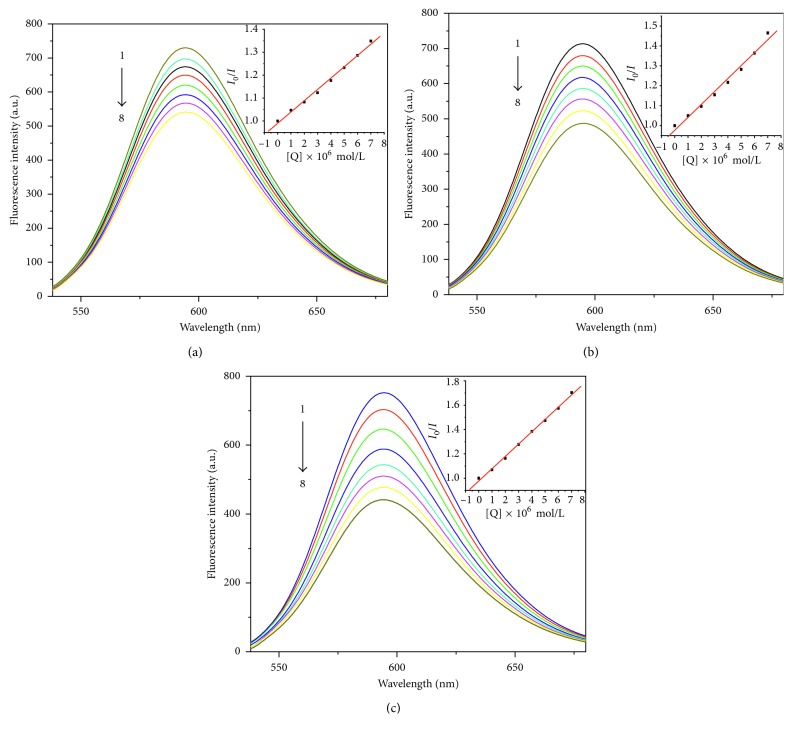
Fluorescence quenching spectra of EB bound to CT-DNA by complexes **1** (a), **2** (b), and **3** (c). λ_ex_ = 510 nm; DNA and EB concentrations were 1.0 × 10^−5^ and 1.0 × 10^−5^ M, and the complex concentrations were 0, 1.0, 2.0, 3.0, 4.0, 5.0, 6.0, and 7.0 × 10^−5^ M corresponding to the curves from 1 to 8, respectively. The arrow shows the intensity change on increasing the complex concentration.

**Figure 6:**
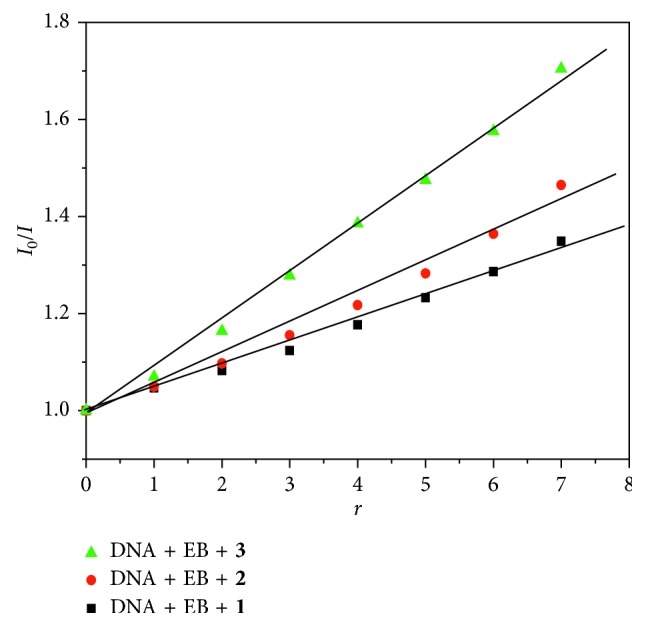
The Stern–Volmer fluorescence quenching curves.

Ethidium bromide (EB) is a classical embedded fluorescent probe that studies the interaction of drug molecules with DNA. It is weakly fluorescent by itself, but the chromophore is embedded in base pairs of DNA molecules to make fluorescence intensity enhancement. When a complex is added to the EB-DNA system, it can interact with DNA and compete with EB for DNA binding, resulting in a decrease in the fluorescence intensity of the EB-DNA system. Therefore, we can deduce the interaction mode of the complex with DNA by observing the degree of change of the fluorescence system. As shown in Figure 5, the fluorescence intensities of the EB-DNA system at 595 nm decreased significantly with the increase of the concentration of the three complexes, indicating that the complexes can displace EB from the CT-DNA by competitive binding. In order to quantitatively study the fluorescence quenching degree of the complexes, the fluorescence quenching constant *K*_sq_ can be calculated by the Stern–Volmer equation [44]:

(2) $$\begin{array}{c} \frac{I_{0}}{I}=1+K_{\mathrm{sq}}\cdot r, \end{array}$$

where *I*_0_ is the fluorescence intensity of the EB-DNA system when the complex is not added, *I* is the fluorescence intensity of the EB-DNA system after adding different concentration of the complexes, *K*_sq_ is the linear Stern–Volmer constant, and *r* ([complex]/[CT-DNA]) is the ratio of concentration of the complex to that of CT-DNA. The Stern–Volmer fluorescence quenching curves are shown in Figure 6. The *K*_sq_ values for these complexes were determined: *K*_sq_(**1**) = 0.493, *K*_sq_(**2**) = 0.648, and *K*_sq_(**3**) = 1.011, respectively.

#### 3.3.3. CD Spectroscopy

**Figure 7:**
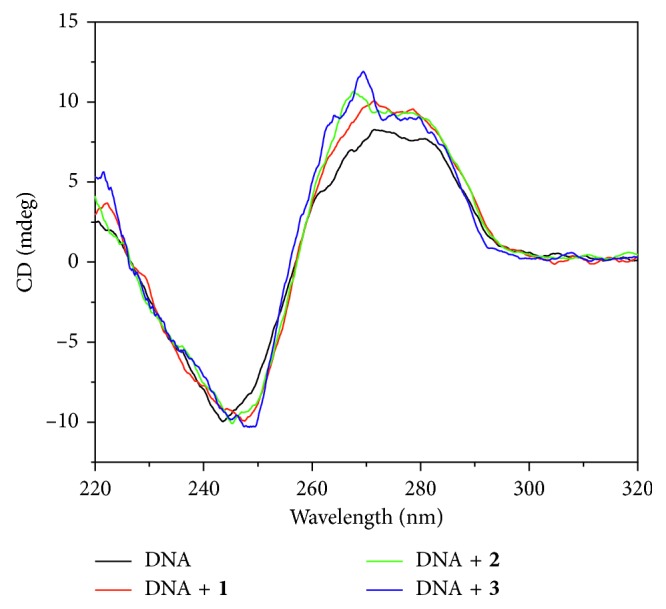
Effect of complexes **1**, **2**, and **3** on CD spectra of CT-DNA. DNA concentration was 1.0 × 10^−4^ M, and the complex concentration was 4.0 × 10^−5^ M.

CD spectroscopy is one of the most important means to study the interaction of small molecules with DNA and explore the change of DNA conformation by measuring the difference in the absorption of left and right circularly polarized light [45]. As shown in Figure 7, there were two distinct peaks at 273 nm and 245 nm in the CD spectrum of CT-DNA. The positive peak at 273 nm was caused by the *π*-*π* stacking of the CT-DNA base pair and the negative peak at 245 nm was due to the helicity, which are typical CD spectra of B-DNA conformation [46]. When the three complexes were added to DNA solution, respectively, each CD spectrum of DNA changed obviously. The positive peak at 273 nm increased, and the negative peak at 245 nm remained unchanged. The degree of positive peak variation by complex **3** is stronger than that of complex **2** and complex **1**. The results indicated that these complexes were inserted into the base pair of DNA. This change affects the *π*-*π* stacking between the DNA base pairs and makes the conformation of DNA changed. This result is consistent with that of the UV-Vis absorption spectra and the fluorescence spectra.

#### 3.3.4. Viscosity Measurement

**Figure 8:**
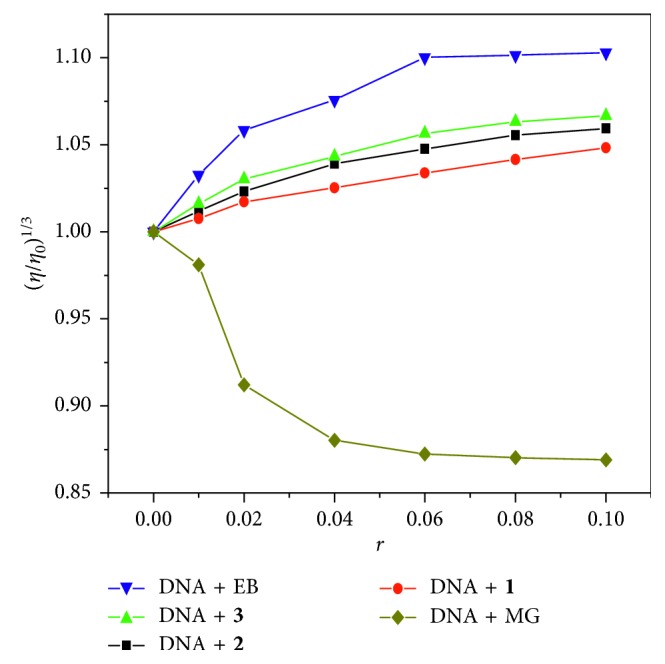
Effect of increasing amounts of complexes **1**, **2**, and **3**, EB, and methyl green on the relative viscosity of CT-DNA (1.0 × 10^−4^ M at pH = 7.4).

Viscosity determination is a unique method for determining the binding mode of the complex to DNA. The distance between adjacent base pairs increases when the complex interacts with DNA, and the insertion process of the small molecules of the complex is achieved. The double helix of the DNA is elongated and the viscosity increases. In contrast, the double-stranded structure of DNA is kinked and the viscosity is reduced when the complex is inserted into the base pair of DNA by partial insertion or groove bonding, while the effect of the groove surface mode or electrostatic action has a smaller effect on viscosity. The changes of the viscosity of DNA solution with increasing concentrations of the complexes are shown in Figure 8. With the increase of the amounts of these complexes, the relative viscosities of DNA solutions increased, which were consistent with the change of viscosity of DNA solution caused by intercalator EB and contrary to the trend of methylene green (MG). The results also indicated that the interaction between complex **3** and DNA is slightly stronger than that of complexes **2** and **1**. This is consistent with the results of the abovementioned spectral studies.

From the abovementioned results, we can conclude that the DNA binding ability of these three complexes is in the order of complex **3** > complex **2** > complex **1**. The difference among the three complexes is using different aldehydes for synthesizing Schiff bases, salicylaldehyde for complex **1**, 2-hydroxy-1-naphthaldehyde for complex **2**, and *o*-vanillin for complex **3**. In 2-hydroxy-1-naphthaldehyde, the benzene group replaces the hydrogen on the benzene ring of salicylaldehyde, and the methoxy group replaces the hydrogen on the benzene ring of salicylaldehyde in *o*-vanillin. As we know, the methoxy group and benzene group are electron-donating groups, and the electron-donating ability of the methoxy group is bigger than that of the benzene group. Therefore, the Schiff base ligand provides electrons more easily to coordinate to Ni(II) in complex **3** than that in complex **2** and complex **1**; thus, the second ligand (1,10-phenanthroline) has higher electron density in complex **3** than that in complex **2** and complex **1**. The interaction of the complexes with DNA is mainly by intercalation of the planar 1,10-phenanthroline ligand into the DNA base pairs, producing the strong *π*-*π* stacking interaction. Complex **3** with higher electron density of the 1,10-phenanthroline ligand can interact with DNA stronger than complex **1** and complex **2**.

### 3.4. SOD Activity

**Figure 9:**
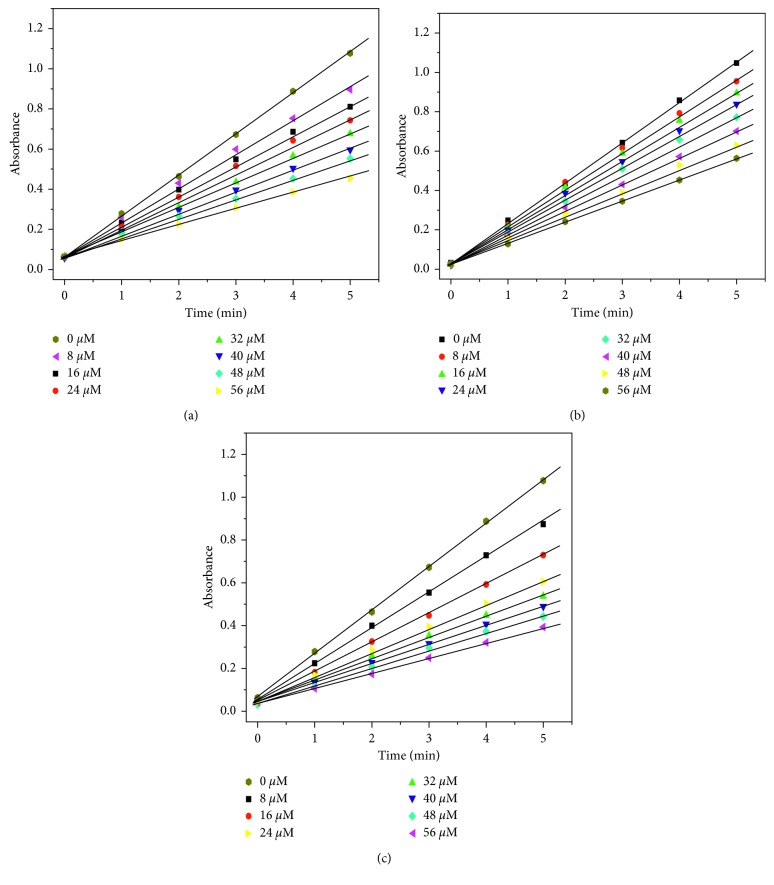
Effect of the concentrations of complexes **1** (a), **2** (b), and **3** (c) on the absorbance (*A*_560_) of the reaction system with time. [NBT] = 1.0 × 10^−4^ mol·L^−1^, [VB_2_] = 6.2 × 10^−6^ mol·L^−1^, [TMEDA] = 8.3 × 10^−4^ mol·L^−1^, [complex] = 0, 0.8 × 10^−5^, 1.6 × 10^−5^, 2.4 × 10^−5^, 3.2 × 10^−5^, 4.0 × 10^−5^, 4.8 × 10^−5^, and 5.6 × 10^−5^ mol·L^−1^.

**Figure 10:**
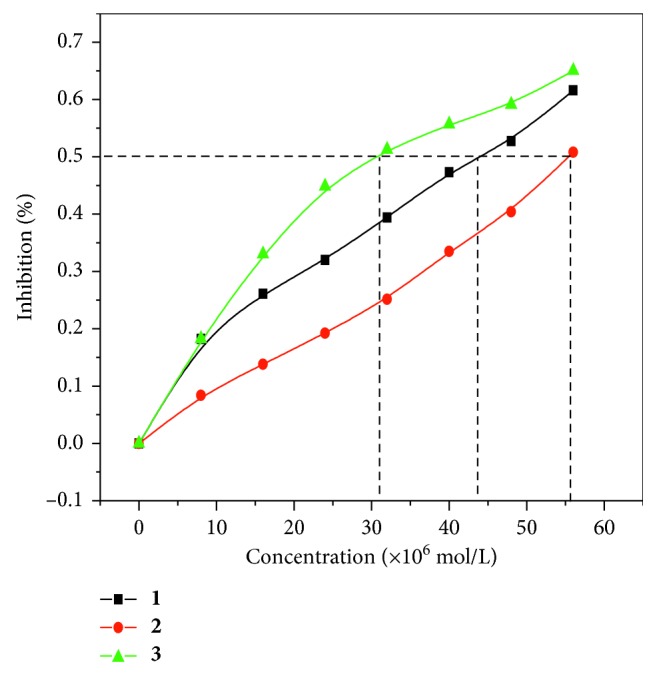
The inhibitory rates of complexes **1**, **2**, and **3** varying with concentration.

The nitrotetrazolium blue chloride (NBT) light reduction method is a commonly used method to determine the activity of SOD. Riboflavin (VB_2_) reacted with tetramethylethylenediamine to produce the superoxide anion radical under light conditions: VB_2_ + (CH_3_)_2_NCH_2_CH_2_N(CH_3_)_2_ → O_2_^·−^. The superoxide anion radical can reduce NBT to a blue-violet compound named formazan. The absorbance at 560 nm was proportional to the concentration of formazan. Therefore, a straight line can be obtained by measuring the absorbance *A*_560_ at different times. The slope of the line (*k* value) can reflect the speed of generation of formazan.  Figure 9 shows that, with the increase of the concentration of the complex, the slope of the line gradually decreased, indicating that the greater the concentration, the greater the inhibition rate. The inhibitory rate (*η*) of the complex to O_2_^·−^ can be determined by the following formula [47]:

(3) $$\begin{array}{c} \eta =\left(1-\frac{k^{\prime }}{k}\right)\times 100\%, \end{array}$$

where *k* is the slope of the straight line without complex and *k*′ is the slope of the straight line after adding the complex. The 50% of activity (IC_50_) indicates the molar concentration of the tested complex which caused a 50% scavenging effect on superoxide radicals.  Figure 10 shows the inhibition rate of the complex to O_2_^·−^ at various concentrations of complexes **1**, **2**, and **3**. The obtained IC_50_ values from Figure 10 were 4.4 × 10^−5^ M for complex **1**, 5.6 × 10^−5^ M for complex **2**, and 3.1 × 10^−5^ M for complex **3**, respectively. The results showed that these complexes have a certain SOD activity, and the superoxide anion radical scavenging effect of complex **3** is higher than that of complex **1** and complex **2**.

## 4. Conclusion

Three new nickel(II) complexes, [Ni(sal-L-phe)(phen)(CH_3_OH)]·CH_3_OH (**1**), [Ni(naph-L-phe)(phen)(CH_3_OH)] (**2**), and [Ni(*o*-van-L-phe)(phen)(CH_3_OH)]·5CH_3_OH (**3**), have been synthesized. These crystal structures were determined by single-crystal X-ray diffraction and characterized by elemental analysis and IR spectra. The interactions of these complexes with CT-DNA were studied by spectroscopies. The results showed that these complexes could interact with CT-DNA by the way of insertion into the CT-DNA, which was due to the good planarity of the ligand 1,10-phenanthroline in these complexes. Furthermore, the SOD activities of these complexes were studied by the NBT photoreduction method, implying that the three complexes had some SOD activities. The investigation can help us understand their mechanism of action and provide basis for designing new transition metal complexes with better activities.

## Figures and Tables

**Table 1:** Crystallographic and structure refinement data for complexes **1**, **2**, and **3**.

Complex	**1**	**2**	**3**
Empirical formula	C_30_H_29_N_3_O_5_Ni	C_33_H_27_N_3_O_4_Ni	C_95_H_101_N_9_O_20_Ni_3_
Formula weight	570.27	588.28	1864.98
Wavelength (Å)	0.71073	0.71073	0.71073
Crystal system	Monoclinic	Triclinic	Triclinic
Space group	C2/c	P-1	P-1
*a* (Å)	26.741(3)	10.8240(8)	12.7068(10)
*b* (Å)	18.1651(17)	11.9041(9)	13.5015(14)
*c* (Å)	11.8350(9)	13.0879(11)	29.9214(17)
*α* (°)	90	67.226(10)	83.667(2)
*β* (°)	108.528(2)	73.566(2)	82.7470(10)
*γ* (°)	90	64.094(10)	63.9790(10)
*v* (Å^3^)	5450.9(8)	1385.43(19)	4567.4(6)
Z	8	2	2
*D*_calc_ (Mg·m^−3^)	1.390	1.410	1.356
*F*(000)	2384	612	1956
Absorption coefficient (mm^−1^)	0.756	0.744	0.687
Crystal size (mm)	0.43 × 0.38 × 0.35	0.23 × 0.20 × 0.12	0.25 × 0.21 × 0.18
*θ* range	2.66 to 25.02	2.19 to 25.01	2.57 to 25.02
Index ranges	–25 ≤ *h* ≤ 31	–12 ≤ *h* ≤ 12	–15 ≤ *h* ≤ 10
	–21 ≤ *k* ≤ 21	–14 ≤ *k* ≤ 11	–16 ≤ *k* ≤ 16
	–13 ≤ *l* ≤ 14	–15 ≤ *l* ≤ 13	–35 ≤ *l* ≤ 35
Reflections collected	13562	7109	29803
Unique reflections	4816	4791	16112
*R*_int_	0.0432	0.0335	0.1085
Max/min transmission	0.7777, 0.7368	0.9160, 0.8475	0.8863, 0.8470
Data, restraint, parameters	4816, 3, 366	4791, 0, 363	16112, 2613, 1155
Goodness-of-fit on *F* ^2^	1.094	0.952	1.010
Final *R* indices (*I* > 2*σ* (*I*))	*R*_1_ = 0.0432, *wR*_2_ = 0.0946	*R*_1_ = 0.0566, *wR*_2_ = 0.1350	*R*_1_ = 0.0867, *wR*_2_ = 0.1790
*R* indices (all data)	*R*_1_ = 0.0930, *wR*_2_ = 0.1248	*R*_1_ = 0.0820, *wR*_2_ = 0.1456	*R*_1_ = 0.1954, *wR*_2_ = 0.2540
Largest diff. peak and hole (e·Å^−3^)	0.392, −0.287	1.099, −0.500	0.788, −0.500

**Table 2:** Selected bond lengths (Å) and bond angles (°) for complexes **1**, **2**, and **3**.

Complex **1**					
Ni(1)–O(3)	1.990(2)	Ni(1)–N(1)	1.991(3)	Ni(1)–O(1)	2.068(3)
Ni(1)–N(3)	2.075(3)	Ni(1)–N(2)	2.116(3)	Ni(1)–O(4)	2.117(3)
O(3)–Ni(1)–N(1)	92.43(11)	O(3)–Ni(1)–O(1)	173.63(10)	N(1)–Ni(1)–O(1)	81.29(11)
O(3)–Ni(1)–N(3)	93.55(11)	N(1)–Ni(1)–N(3)	172.77(11)	O(1)–Ni(1)–N(3)	92.64(11)
O(3)–Ni(1)–N(2)	91.84(11)	N(1)–Ni(1)–N(2)	96.26(12)	O(1)–Ni(1)–N(2)	87.84(10)
N(3)–Ni(1)–N(2)	79.50(12)	O(3)–Ni(1)–O(4)	89.99(11)	N(1)–Ni(1)–O(4)	92.72(11)
O(1)–Ni(1)–O(4)	91.34(11)	N(3)–Ni(1)–O(4)	91.33(11)	N(2)–Ni(1)–O(4)	170.75(11)

Complex **2**					
Ni(1)–N(1)	1.987(3)	Ni(1)–O(3)	1.997(3)	Ni(1)–O(1)	2.068(3)
Ni(1)–N(3)	2.083(3)	Ni(1)–O(4)	2.100(3)	Ni(1)–N(2)	2.129(3)
N(1)–Ni(1)–O(3)	89.99(12)	N(1)–Ni(1)–O(1)	81.80(12)	O(3)–Ni(1)–O(1)	171.26(10)
N(1)–Ni(1)–N(3)	175.09(12)	O(3)–Ni(1)–N(3)	94.34(11)	O(1)–Ni(1)–N(3)	93.75(11)
N(1)–Ni(1)–O(4)	90.64(13)	O(3)–Ni(1)–O(4)	92.52(12)	O(1)–Ni(1)–O(4)	90.56(12)
N(3)–Ni(1)–O(4)	91.50(13)	N(1)–Ni(1)–N(2)	98.70(12)	O(3)–Ni(1)–N(2)	90.17(11)
O(1)–Ni(1)–N(2)	88.13(11)	N(3)–Ni(1)–N(2)	78.99(12)	O(4)–Ni(1)–N(2)	170.29(12)

Complex **3**					
Ni(1)–N(1)	2.000(6)	Ni(1)–O(3)	2.003(5)	Ni(1)–O(1)	2.053(5)
Ni(1)–N(3)	2.076(6)	Ni(1)–N(2)	2.131(6)	Ni(1)–O(5)	2.146(5)
Ni(2)–N(4)	1.982(6)	Ni(2)–O(8)	1.992(5)	Ni(2)–O(6)	2.059(5)
Ni(2)–N(6)	2.075(7)	Ni(2)–O(10)	2.113(5)	Ni(2)–N(5)	2.116(6)
Ni(3)–N(7)	1.995(7)	Ni(3)–O(13)	2.008(5)	Ni(3)–O(11)	2.056(6)
Ni(3)–N(8)	2.084(7)	Ni(3)–O(15)	2.099(6)	Ni(3)–N(9)	2.114(7)
N(1)–Ni(1)–O(3)	92.3(2)	N(1)–Ni(1)–O(1)	82.0(2)	O(3)–Ni(1)–O(1)	173.3(2)
N(1)–Ni(1)–N(3)	169.9(2)	O(3)–Ni(1)–N(3)	93.2(2)	O(1)–Ni(1)–N(3)	92.9(2)
N(1)–Ni(1)–N(2)	92.6(2)	O(3)–Ni(1)–N(2)	96.1(2)	O(1)–Ni(1)–N(2)	87.8(2)
N(3)–Ni(1)–N(2)	78.4(2)	N(1)–Ni(1)–O(5)	95.3(2)	O(3)–Ni(1)–O(5)	90.31(18)
O(1)–Ni(1)–O(5)	86.70(19)	N(3)–Ni(1)–O(5)	93.2(2)	N(2)–Ni(1)–O(5)	169.7(2)
N(4)–Ni(2)–O(8)	92.7(2)	N(4)–Ni(2)–O(6)	81.3(2)	O(8)–Ni(2)–O(6)	174.0(2)
N(4)–Ni(2)–N(6)	171.7(2)	O(8)–Ni(2)–N(6)	93.3(2)	O(6)–Ni(2)–N(6)	92.7(2)
N(4)–Ni(2)–O(10)	93.9(2)	O(8)–Ni(2)–O(10)	90.41(19)	O(6)–Ni(2)–O(10)	89.3(2)
N(6)–Ni(2)–O(10)	91.6(2)	N(4)–Ni(2)–N(5)	95.0(2)	O(8)–Ni(2)–N(5)	90.8(2)
O(6)–Ni(2)–N(5)	90.5(2)	N(6)–Ni(2)–N(5)	79.3(3)	O(10)–Ni(2)–N(5)	170.9(3)
N(7)–Ni(3)–O(13)	91.9(2)	N(7)–Ni(3)–O(11)	80.3(3)	O(13)–Ni(3)–O(11)	171.9(2)
N(7)–Ni(3)–N(8)	169.6(3)	O(13)–Ni(3)–N(8)	96.3(2)	O(11)–Ni(3)–N(8)	91.6(2)
N(7)–Ni(3)–O(15)	94.9(3)	O(13)–Ni(3)–O(15)	89.3(2)	O(11)–Ni(3)–O(15)	89.1(2)
N(8)–Ni(3)–O(15)	91.6(3)	N(7)–Ni(3)–N(9)	95.4(3)	O(13)–Ni(3)–N(9)	91.4(2)
O(11)–Ni(3)–N(9)	91.6(2)	N(8)–Ni(3)–N(9)	78.1(3)	O(15)–Ni(3)–N(9)	169.6(3)

**Table 3:** Hydrogen bond lengths (Å) and bond angles (°) for complexes **1**, **2**, and **3**.

D−H⋯A	*d*(D−H)	*d*(H−A)	*d*(D−A)	∠(DHA)
Complex **1**				
O4–H4⋯O2^#1^	0.82	1.79	2.609(4)	173.4
C26–H26⋯O4	0.93	2.60	3.141(5)	117.9
O(5′)–H(5A′)⋯O(2)	0.82	2.15	3.175(5)	136.8
O(5)–H(5A)⋯O(2)	0.82	2.02	2.765(8)	149.9

Complex **2**				
O4–H4⋯O2^#1^	0.82	1.76	2.576(4)	170.4
C32–H32⋯O2^#3^	0.93	2.36	3.274(5)	166.8
Complex **3**				

O(16)–H(16)⋯O(2)	0.82	2.03	2.828(10)	163.2
O(15)–H(15)⋯O(18)^#4^	0.82	1.83	2.640(10)	171.2
O(5)–H(5)⋯O(7)^#5^	0.82	1.93	2.697(8)	154.2
O(10)–H(10)⋯O(12)^#5^	0.82	1.78	2.588(8)	169.9
O(17)–H(17)⋯O(2)^#6^	0.82	2.00	2.733(9)	148.3
O(19)–H(19)⋯O(7)^#6^	0.82	2.03	2.853(9)	177.6
O(18)–H(18)⋯O(11)^#6^	0.82	1.86	2.659(9)	166.1
O(20)–H(20)⋯O(13)^#7^	0.82	2.06	2.884(12)	179.0
O(20)–H(20)⋯O(14)^#7^	0.82	2.55	2.966(12)	112.5

Symmetry codes for the complex: #1: 0.5−*x*, 0.5−*y*, and 1−*z*; #2: 2−*x*, −*y*, and 1−*z*; #3: 1−*x*, −*y*, and 1−*z*; #4: −*x*+1, −*y* + 1, and −*z*; #5: −*x* + 2, −*y* + 1, and −*z* + 1; #6: *x*−1, *y*, and *z*; #7: *x*, *y*, and *z* + 1.

## Data Availability

The data used to support the findings of this study are available from the corresponding author upon request.

## Supplementary Materials

Supplementary Materials Figure S1: mass spectrum of the Schiff base **1**. Figure S2: mass spectrum of the Schiff base **2**. Figure S3: mass spectrum of the Schiff base **3**. Figure S4: ^1^H NMR spectrum of the Schiff base **1**. Figure S5: ^1^H NMR spectrum of the Schiff base **2**. Figure S6: ^1^H NMR spectrum of the Schiff base **3**. CheckCIF/PLATON reports for the crystal structure determinations of complexes **1**, **2**, and **3**.
