# Supplementary material for: Synthesis, Structure, DNA Interaction, and SOD Activity of Three Nickel(II) Complexes Containing L-Phenylalanine Schiff Base and 1,10-Phenanthroline
Source: Bioinorg Chem Appl. 2018 Jul 5;2018:8478152. doi: 10.1155/2018/8478152 (PMC6057355; doi:10.1155/2018/8478152)

Supplementary Materials:

1. Mass spectra of the Schiff bases 1, 2 and 3

The mass spectra showed ion peak at m/z = 270.11 as the molecular ion peak ($[M+H]^+$) of Schiff base **1** (Fig. S1). The ion peak at m/z = 320.13 is due to the molecular ion peak ($[M+H]^+$) of Schiff base **2** (Fig. S2). The ion peak at m/z = 300.12 corresponds to the molecular ion peak ($[M+H]^+$) of Schiff base **3** (Fig. S3). The above results are consistent with the theoretical molecular weight of Schiff base **1** ($C_{16}H_{15}NO_3$: Mr = 269.30), Schiff base **2** ($C_{20}H_{17}NO_3$: Mr = 319.36) and Schiff base **3** ($C_{17}H_{17}NO_4$: Mr = 299.32), respectively.

Schiff base **1**

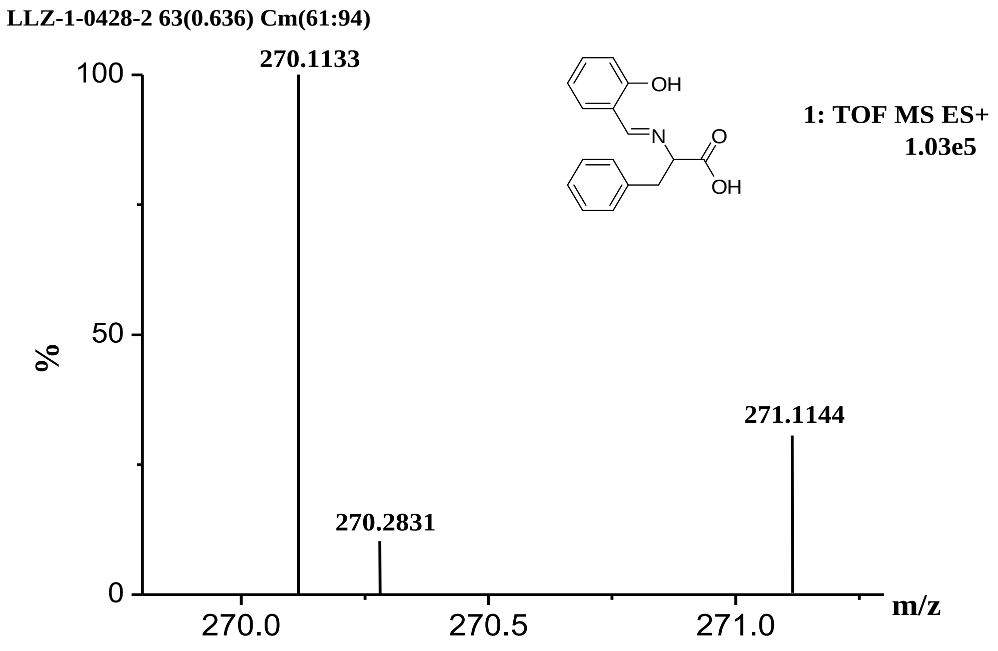

Fig. S1. Mass spectrum of the Schiff base **1**

Schiff base **2**

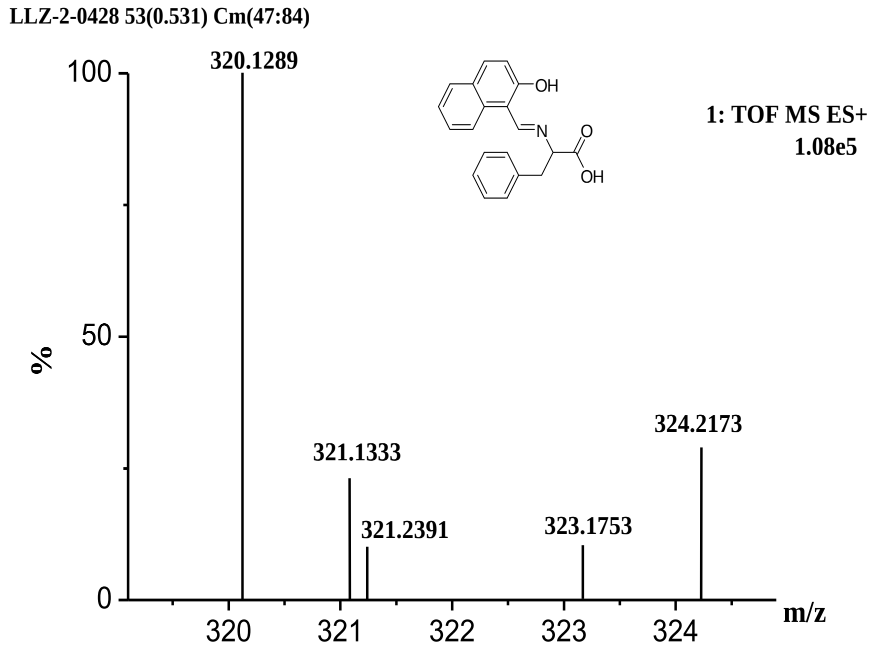

Fig. S2. Mass spectrum of the Schiff base **2**

Schiff base **3**

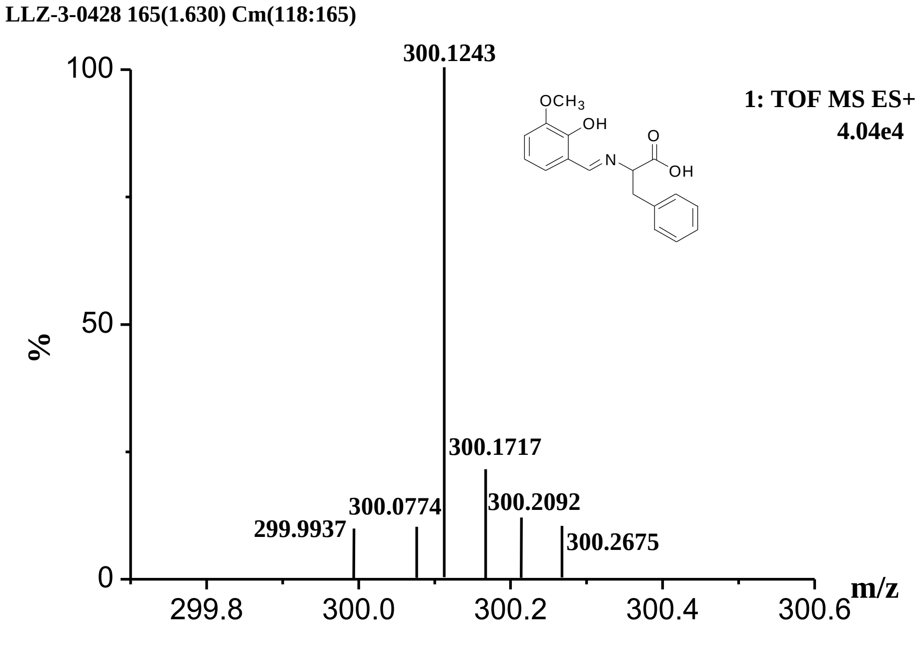

Fig. S3. Mass spectrum of the Schiff base **3**

2. ^{1}H NMR spectra of the Schiff bases 1, 2 and 3

The ^{1}H NMR spectra of the Schiff bases **1**, **2** and **3** in dimethyl sulfoxide (DMSO-d_6) were recorded on Varian 400 MHz spectrometer. ^{1}H NMR spectra of Schiff bases **1** (Fig. S4), **2** (Fig. S5) and **3** (Fig. S6) were assigned as follows.

Schiff base **1**:

δ: 14.30 (s, 1H, COOH), 8.09 (s, 1H, CH=N), 7.20 (t, *J*=7.6, 4H, ArH), 7.15 (d, *J*=6.8, 2H, ArH), 7.10 (t, *J*=7.0, 1H, ArH), 6.71 (d, *J*=8.1, 1H, ArH), 6.66 (t, *J*=7.4, 1H, ArH), 3.82 (dd, *J*=9.5, 3.9, 1H, CH), 3.28 (dd, *J*=13.8, 3.9, 1H, CH_2), 2.92 (dd, *J*=13.8, 9.5, 1H, CH_2).

Schiff base **2:**

δ: 13.46 (s, 1H, COOH), 8.36 (d, *J*=12.5, 1H, CH=N), 7.62 – 7.55 (m, 2H, ArH), 7.51 (d, *J*=7.4, 1H, ArH), 7.23 (d, *J*=17.7, 5H, ArH), 7.12 (s, 1H, ArH), 7.05 (t, *J*=7.3, 1H, ArH), 6.57 (d, *J*=9.4, 1H, ArH), 4.12 (s, 1H, CH), 2.96 – 2.83 (m, 1H, CH_2).

Schiff base **3**:

δ: 14.23 (s, 1H, COOH), 7.95 (s, 1H, CH=N), 7.23 – 7.17 (m, 2H, ArH), 7.17 – 7.09 (m, 3H, ArH), 6.78 (d, *J*=6.4, 1H, ArH), 6.69 (dd, *J*=8.0, 1.4, 1H, ArH), 6.41 (t, *J*=7.8, 1H, ArH), 3.87 (d, *J*=9.1, 1H, CH), 3.70 (s, 3H, OCH_3), 3.28 (dd, *J*=14.0, 3.8, 1H, CH_2), 2.91 (dd, *J*=13.9, 9.7, 1H, CH_2).

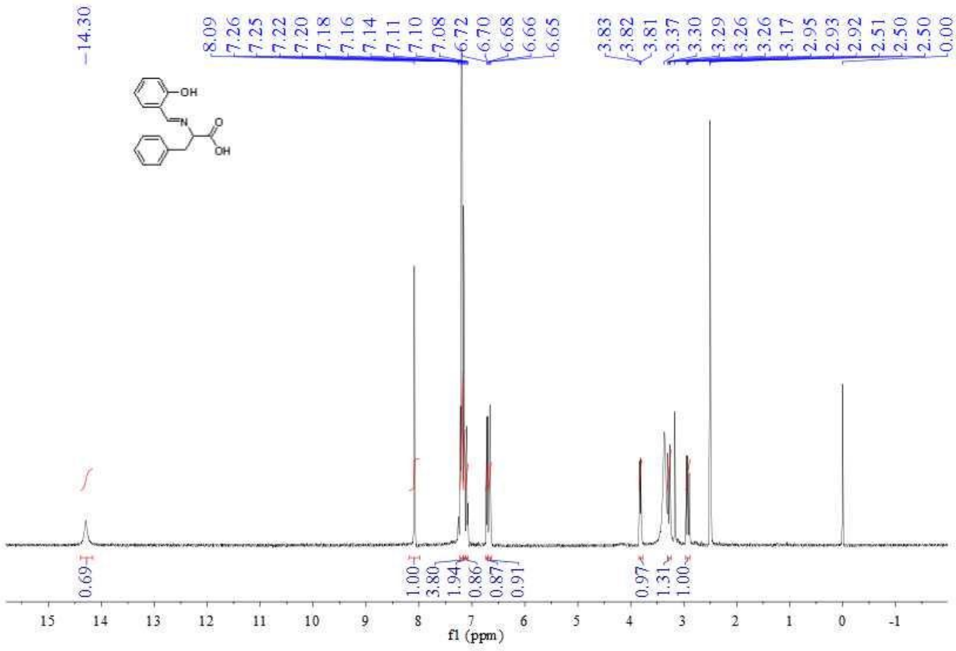

Fig. S4. ^{1}H NMR spectrum of the Schiff base **1**

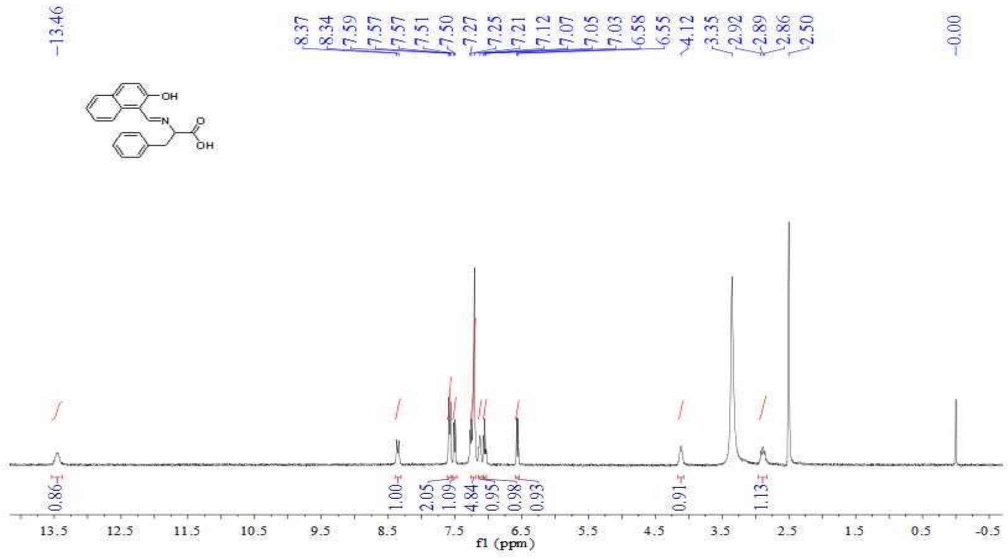

Fig. S5. ^{1}H NMR spectrum of the Schiff base **2**

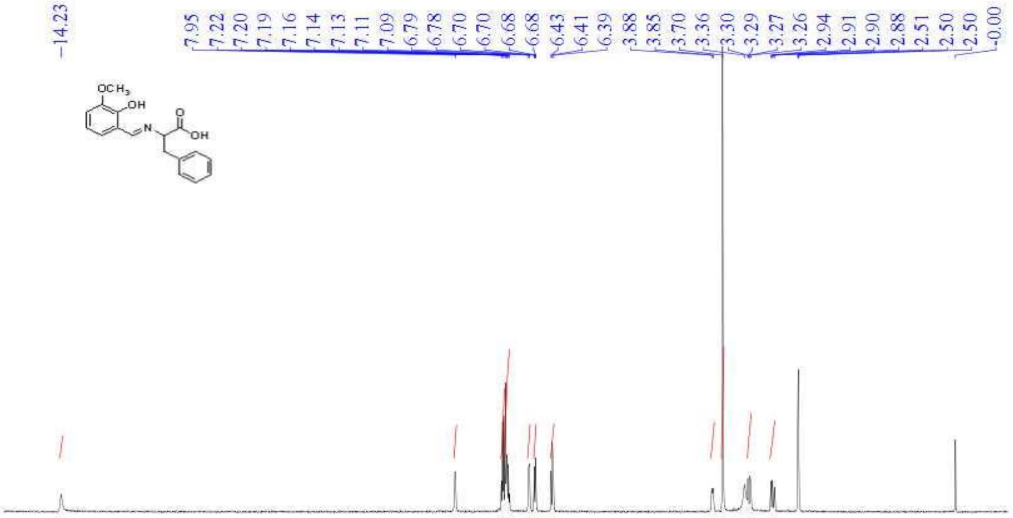

Fig. S6. ^{1}H NMR spectrum of the Schiff base **3**

3. CheckCIF/PLATON reports for the crystal structure determinations of the Complexes 1, 2 and 3

3.1 Complex **1**

checkCIF/PLATON report

You have not supplied any structure factors. As a result the full set of tests cannot be run.

THIS REPORT IS FOR GUIDANCE ONLY. IF USED AS PART OF A REVIEW PROCEDURE FOR PUBLICATION, IT SHOULD NOT REPLACE THE EXPERTISE OF AN EXPERIENCED CRYSTALLOGRAPHIC REFEREE.

No syntax errors found. CIF dictionary Interpreting this report

Datablock: 131107e

```
Bond precision:  C-C = 0.0062 A          Wavelength=0.71073

Cell:            a=26.741(3)    b=18.1651(17)       c=11.8350(9)
                 alpha=90       beta=108.528(2)     gamma=90
Temperature:     298 K

                 Calculated                 Reported
Volume           5450.9(9)                  5450.9(8)
Space group      C 2/c                      C2/c
Hall group       -C 2yc                     ?
Moiety formula   C29 H25 N3 Ni O4, C H4 O   ?
Sum formula      C30 H29 N3 Ni O5           C30 H29 N3 Ni O5
Mr               570.25                     570.27
Dx,g cm-3        1.390                      1.390
Z                8                          8
Mu (mm-1)        0.756                      0.756
F000             2384.0                     2384.0
F000'            2387.64
h,k,lmax         31,21,14                   31,21,14
Nref             4824                       4816
Tmin,Tmax        0.730,0.768                0.737,0.778
Tmin'            0.715

Correction method= # Reported T Limits: Tmin=0.737 Tmax=0.778
AbsCorr = MULTI-SCAN

Data completeness= 0.998          Theta(max)= 25.020

R(reflections)= 0.0432( 3035)     wR2(reflections)= 0.1248( 4816)

S = 1.094                 Npar= 366
```

```
The following ALERTS were generated. Each ALERT has the format
      test-name_ALERT_alert-type_alert-level.
Click on the hyperlinks for more details of the test.
```

Alert level C

```
PLAT331_ALERT_2_C Small Average Phenyl C-C Dist C4       -C9             1.37 Ang.
PLAT341_ALERT_3_C Low Bond Precision on  C-C Bonds ...............    0.00625 Ang.
PLAT480_ALERT_4_C Long H...A H-Bond Reported H29A     ..O3               2.63 Ang.
```

Alert level G

```
PLAT002_ALERT_2_G Number of Distance or Angle Restraints on AtSite          4 Note
PLAT005_ALERT_5_G No Embedded Refinement Details Found  in the CIF     Please Do !
PLAT007_ALERT_5_G Number of Unrefined Donor-H Atoms ..............          3 Report
PLAT083_ALERT_2_G SHELXL Second Parameter in WGHT  Unusually Large       5.89 Why ?
PLAT093_ALERT_1_G No s.u.'s on H-positions, Refinement Reported as      mixed Check
PLAT128_ALERT_4_G Alternate Setting for Input Space Group     C2/c       I2/a Note
PLAT302_ALERT_4_G Anion/Solvent/Minor-Residue Disorder (Resd  2  )       100% Note
PLAT302_ALERT_4_G Anion/Solvent/Minor-Residue Disorder (Resd  3  )       100% Note
PLAT304_ALERT_4_G Non-Integer Number of Atoms in ...... Resd  2          3.17 Check
PLAT304_ALERT_4_G Non-Integer Number of Atoms in ...... Resd  3          2.83 Check
PLAT720_ALERT_4_G Number of Unusual/Non-Standard Labels ..........          1 Note
PLAT793_ALERT_4_G Model has Chirality at C2           (Centro SPGR)         R Verify
PLAT860_ALERT_3_G Number of Least-Squares Restraints .............          3 Note
PLAT899_ALERT_4_G SHELXL97   is Deprecated and Succeeded by SHELXL       2018 Note
```

```
   0 ALERT level A = Most likely a serious problem - resolve or explain
   0 ALERT level B = A potentially serious problem, consider carefully
   3 ALERT level C = Check. Ensure it is not caused by an omission or oversight
  14 ALERT level G = General information/check it is not something unexpected

   1 ALERT type 1 CIF construction/syntax error, inconsistent or missing data
   3 ALERT type 2 Indicator that the structure model may be wrong or deficient
   2 ALERT type 3 Indicator that the structure quality may be low
   9 ALERT type 4 Improvement, methodology, query or suggestion
   2 ALERT type 5 Informative message, check
```

It is advisable to attempt to resolve as many as possible of the alerts in all categories. Often the minor alerts point to easily fixed oversights, errors and omissions in your CIF or refinement strategy, so attention to these fine details can be worthwhile. In order to resolve some of the more serious problems it may be necessary to carry out additional measurements or structure refinements. However, the purpose of your study may justify the reported deviations and the more serious of these should normally be commented upon in the discussion or experimental section of a paper or in the "special_details" fields of the CIF. checkCIF was carefully designed to identify outliers and unusual parameters, but every test has its limitations and alerts that are not important in a particular case may appear. Conversely, the absence of alerts does not guarantee there are no aspects of the results needing attention. It is up to the individual to critically assess their own results and, if necessary, seek expert advice.

Publication of your CIF in IUCr journals

A basic structural check has been run on your CIF. These basic checks will be run on all CIFs submitted for publication in IUCr journals (*Acta Crystallographica*, *Journal of Applied Crystallography*, *Journal of Synchrotron Radiation*); however, if you intend to submit to *Acta Crystallographica Section C* or *E* or *IUCrData*, you should make sure that full publication checks are run on the final version of your CIF prior to submission.

Publication of your CIF in other journals

Please refer to the *Notes for Authors* of the relevant journal for any special instructions relating to CIF submission.

PLATON version of 30/01/2018; check.def file version of 30/01/2018

Datablock 131107e - ellipsoid plot

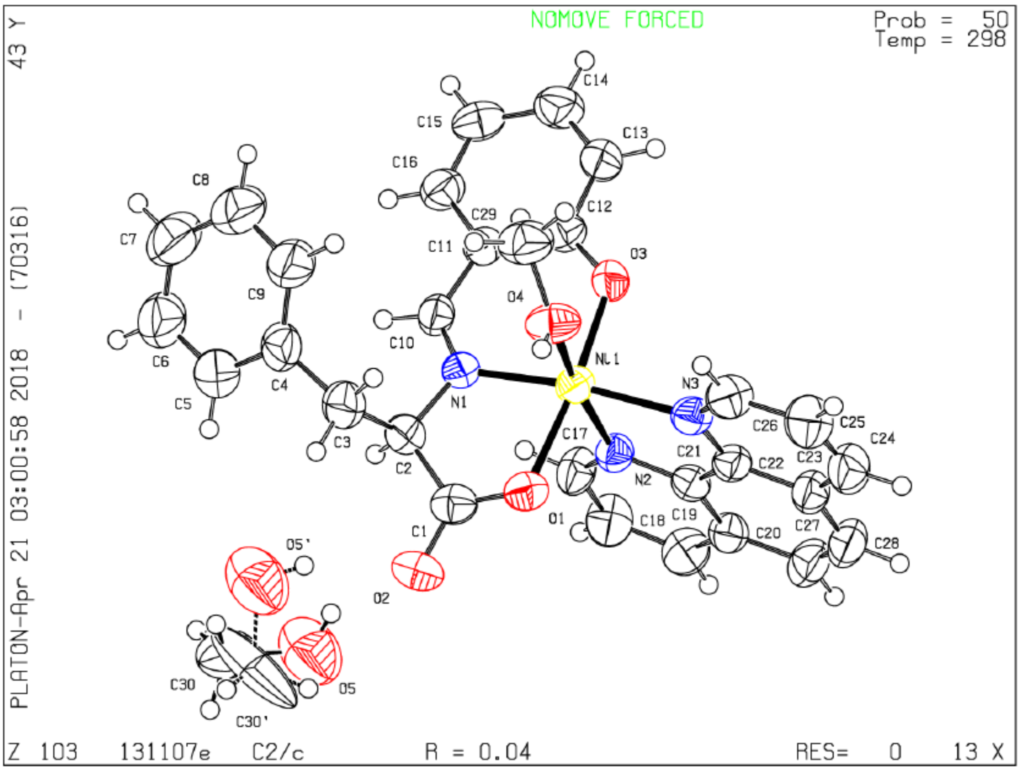

3.2 Complex **2**

checkCIF/PLATON report

You have not supplied any structure factors. As a result the full set of tests cannot be run.

THIS REPORT IS FOR GUIDANCE ONLY. IF USED AS PART OF A REVIEW PROCEDURE FOR PUBLICATION, IT SHOULD NOT REPLACE THE EXPERTISE OF AN EXPERIENCED CRYSTALLOGRAPHIC REFEREE.

No syntax errors found.   CIF dictionary   Interpreting this report

Datablock: 151211e

```
Bond precision:  C-C = 0.0065 A          Wavelength=0.71073

Cell:          a=10.8240(8)      b=11.9041(9)     c=13.0879(11)
               alpha=67.226(1)   beta=73.566(2)   gamma=64.094(1)
Temperature:   298 K

                Calculated                 Reported
Volume          1385.43(19)                1385.43(19)
Space group     P -1                       P -1
Hall group      -P 1                       -P 1
Moiety formula  C33 H27 N3 Ni O4           ?
Sum formula     C33 H27 N3 Ni O4           C33 H27 N3 Ni O4
Mr              588.27                     588.28
Dx,g cm-3       1.410                      1.410
Z               2                          2
Mu (mm-1)       0.744                      0.744
F000            612.0                      612.0
F000'           612.90
h,k,lmax        12,14,15                   12,14,15
Nref            4893                       4791
Tmin,Tmax       0.843,0.915
Tmin'           0.843

Correction method= Not given

Data completeness= 0.979          Theta(max)= 25.015

R(reflections)= 0.0566( 3363)     wR2(reflections)= 0.1456( 4791)

S = 0.952                  Npar= 363
```

The following ALERTS were generated. Each ALERT has the format
test-name_ALERT_alert-type_alert-level.
Click on the hyperlinks for more details of the test.

Alert level C

```
PLAT018_ALERT_1_C _diffrn_measured_fraction_theta_max .NE. *_full          ! Check
PLAT094_ALERT_2_C Ratio of Maximum / Minimum Residual Density ....        2.20 Report
PLAT220_ALERT_2_C Non-Solvent  Resd 1  C   Ueq(max)/Ueq(min) Range         3.8 Ratio
PLAT234_ALERT_4_C Large Hirshfeld Difference C7        --C8               0.16 Ang.
PLAT241_ALERT_2_C High   'MainMol' Ueq as Compared to Neighbors of          C7 Check
PLAT341_ALERT_3_C Low Bond Precision on  C-C Bonds ..............      0.00645 Ang.
PLAT410_ALERT_2_C Short Intra H...H Contact  H10       ..H17              1.98 Ang.
```

Alert level G

```
PLAT793_ALERT_4_G Model has Chirality at C2          (Centro SPGR)          S Verify
```

```
   0 ALERT level A = Most likely a serious problem - resolve or explain
   0 ALERT level B = A potentially serious problem, consider carefully
   7 ALERT level C = Check. Ensure it is not caused by an omission or oversight
   1 ALERT level G = General information/check it is not something unexpected

   1 ALERT type 1 CIF construction/syntax error, inconsistent or missing data
   4 ALERT type 2 Indicator that the structure model may be wrong or deficient
   1 ALERT type 3 Indicator that the structure quality may be low
   2 ALERT type 4 Improvement, methodology, query or suggestion
   0 ALERT type 5 Informative message, check
```

It is advisable to attempt to resolve as many as possible of the alerts in all categories. Often the minor alerts point to easily fixed oversights, errors and omissions in your CIF or refinement strategy, so attention to these fine details can be worthwhile. In order to resolve some of the more serious problems it may be necessary to carry out additional measurements or structure refinements. However, the purpose of your study may justify the reported deviations and the more serious of these should normally be commented upon in the discussion or experimental section of a paper or in the "special_details" fields of the CIF. checkCIF was carefully designed to identify outliers and unusual parameters, but every test has its limitations and alerts that are not important in a particular case may appear. Conversely, the absence of alerts does not guarantee there are no aspects of the results needing attention. It is up to the individual to critically assess their own results and, if necessary, seek expert advice.

Publication of your CIF in IUCr journals

A basic structural check has been run on your CIF. These basic checks will be run on all CIFs submitted for publication in IUCr journals (*Acta Crystallographica*, *Journal of Applied Crystallography*, *Journal of Synchrotron Radiation*); however, if you intend to submit to *Acta Crystallographica Section C* or *E* or *IUCrData*, you should make sure that full publication checks are run on the final version of your CIF prior to submission.

Publication of your CIF in other journals

Please refer to the *Notes for Authors* of the relevant journal for any special instructions relating to CIF submission.

PLATON version of 30/01/2018; check.def file version of 30/01/2018

Datablock 151211e - ellipsoid plot

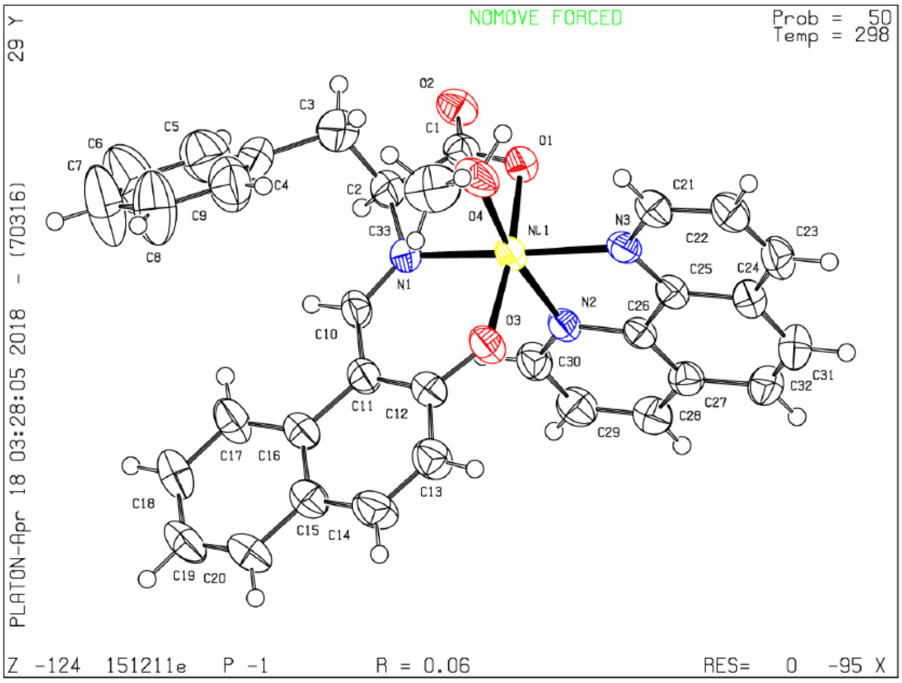

3.3 Complex **3**

checkCIF/PLATON report

You have not supplied any structure factors. As a result the full set of tests cannot be run.

THIS REPORT IS FOR GUIDANCE ONLY. IF USED AS PART OF A REVIEW PROCEDURE FOR PUBLICATION, IT SHOULD NOT REPLACE THE EXPERTISE OF AN EXPERIENCED CRYSTALLOGRAPHIC REFEREE.

No syntax errors found. CIF dictionary Interpreting this report

Datablock: 130323h

```
Bond precision:  C-C = 0.0136 A          Wavelength=0.71073

Cell:           a=12.7068(10)     b=13.5015(14)     c=29.9214(17)
                alpha=83.667(2)   beta=82.747(1)    gamma=63.979(1)
Temperature:    293 K
```

	Calculated	Reported
Volume	4567.4(6)	4567.4(6)
Space group	P -1	P-1
Hall group	-P 1	?
Moiety formula	3(C30 H27 N3 Ni O5), 5(C H4 O)	?
Sum formula	C95 H101 N9 Ni3 O20	C95 H101 N9 Ni3 O20
Mr	1864.92	1864.98
Dx,g cm-3	1.356	1.356
Z	2	2
Mu (mm-1)	0.687	0.687
F000	1956.0	1956.0
F000'	1958.85	
h,k,lmax	15,16,35	15,16,35
Nref	16133	16112
Tmin,Tmax	0.842,0.884	0.847,0.886
Tmin'	0.842	

```
Correction method= # Reported T Limits: Tmin=0.847 Tmax=0.886
AbsCorr = MULTI-SCAN

Data completeness= 0.999          Theta(max)= 25.020

R(reflections)= 0.0870( 6748)     wR2(reflections)= 0.2584( 16112)

S = 1.004                 Npar= 1155
```

The following ALERTS were generated. Each ALERT has the format
test-name_ALERT_alert-type_alert-level.
Click on the hyperlinks for more details of the test.

Alert level C

```
PLAT026_ALERT_3_C Ratio Observed / Unique Reflections (too) Low ..        42% Check
PLAT084_ALERT_3_C High wR2 Value (i.e. > 0.25) ...................       0.26 Report
PLAT220_ALERT_2_C Non-Solvent  Resd 3  C   Ueq(max)/Ueq(min) Range        3.5 Ratio
PLAT230_ALERT_2_C Hirshfeld Test Diff for   O15       --C90       .       5.3 s.u.
PLAT230_ALERT_2_C Hirshfeld Test Diff for   C64       --C69       .       5.9 s.u.
PLAT230_ALERT_2_C Hirshfeld Test Diff for   C81       --C82       .       5.3 s.u.
PLAT234_ALERT_4_C Large Hirshfeld Difference C6        --C7              0.16 Ang.
PLAT234_ALERT_4_C Large Hirshfeld Difference C38       --C39             0.17 Ang.
PLAT234_ALERT_4_C Large Hirshfeld Difference C62       --C63             0.16 Ang.
PLAT241_ALERT_2_C High   'MainMol' Ueq as Compared to Neighbors of        C38 Check
PLAT241_ALERT_2_C High   'MainMol' Ueq as Compared to Neighbors of        C66 Check
PLAT242_ALERT_2_C Low    'MainMol' Ueq as Compared to Neighbors of         O9 Check
PLAT242_ALERT_2_C Low    'MainMol' Ueq as Compared to Neighbors of        O14 Check
PLAT242_ALERT_2_C Low    'MainMol' Ueq as Compared to Neighbors of        O15 Check
PLAT242_ALERT_2_C Low    'MainMol' Ueq as Compared to Neighbors of        C64 Check
PLAT331_ALERT_2_C Small Average Phenyl C-C Dist C34      -C39            1.37 Ang.
PLAT341_ALERT_3_C Low Bond Precision on  C-C Bonds ...............    0.01364 Ang.
```

Alert level G

```
PLAT002_ALERT_2_G Number of Distance or Angle Restraints on AtSite         30 Note
PLAT003_ALERT_2_G Number of Uiso or Uij Restrained non-H Atoms ...        127 Report
PLAT005_ALERT_5_G No Embedded Refinement Details Found  in the CIF     Please Do !
PLAT007_ALERT_5_G Number of Unrefined Donor-H Atoms ..............          8 Report
PLAT066_ALERT_1_G Predicted and Reported Tmin&Tmax Range Identical          ? Check
PLAT199_ALERT_1_G Reported _cell_measurement_temperature ..... (K)        293 Check
PLAT200_ALERT_1_G Reported   _diffrn_ambient_temperature ..... (K)        293 Check
PLAT793_ALERT_4_G Model has Chirality at C2          (Centro SPGR)          R Verify
PLAT793_ALERT_4_G Model has Chirality at C32         (Centro SPGR)          S Verify
PLAT793_ALERT_4_G Model has Chirality at C62         (Centro SPGR)          R Verify
PLAT860_ALERT_3_G Number of Least-Squares Restraints .............       3688 Note
PLAT899_ALERT_4_G SHELXL97   is Deprecated and Succeeded by SHELXL       2018 Note
```

```
   0 ALERT level A = Most likely a serious problem - resolve or explain
   0 ALERT level B = A potentially serious problem, consider carefully
  17 ALERT level C = Check. Ensure it is not caused by an omission or oversight
  12 ALERT level G = General information/check it is not something unexpected

   3 ALERT type 1 CIF construction/syntax error, inconsistent or missing data
  13 ALERT type 2 Indicator that the structure model may be wrong or deficient
   4 ALERT type 3 Indicator that the structure quality may be low
   7 ALERT type 4 Improvement, methodology, query or suggestion
   2 ALERT type 5 Informative message, check
```

It is advisable to attempt to resolve as many as possible of the alerts in all categories. Often the minor alerts point to easily fixed oversights, errors and omissions in your CIF or refinement strategy, so attention to these fine details can be worthwhile. In order to resolve some of the more serious problems it may be necessary to carry out additional measurements or structure refinements. However, the purpose of your study may justify the reported deviations and the more serious of these should normally be commented upon in the discussion or experimental section of a paper or in the "special_details" fields of the CIF. checkCIF was carefully designed to identify outliers and unusual parameters, but every test has its limitations and alerts that are not important in a particular case may appear. Conversely, the absence of alerts does not guarantee there are no aspects of the results needing attention. It is up to the individual to critically assess their own results and, if necessary, seek expert advice.

Publication of your CIF in IUCr journals

A basic structural check has been run on your CIF. These basic checks will be run on all CIFs submitted for publication in IUCr journals (*Acta Crystallographica*, *Journal of Applied Crystallography*, *Journal of Synchrotron Radiation*); however, if you intend to submit to *Acta Crystallographica Section C* or *E* or *IUCrData*, you should make sure that full publication checks are run on the final version of your CIF prior to submission.

Publication of your CIF in other journals

Please refer to the *Notes for Authors* of the relevant journal for any special instructions relating to CIF submission.

PLATON version of 30/01/2018; check.def file version of 30/01/2018

Datablock 130323h - ellipsoid plot

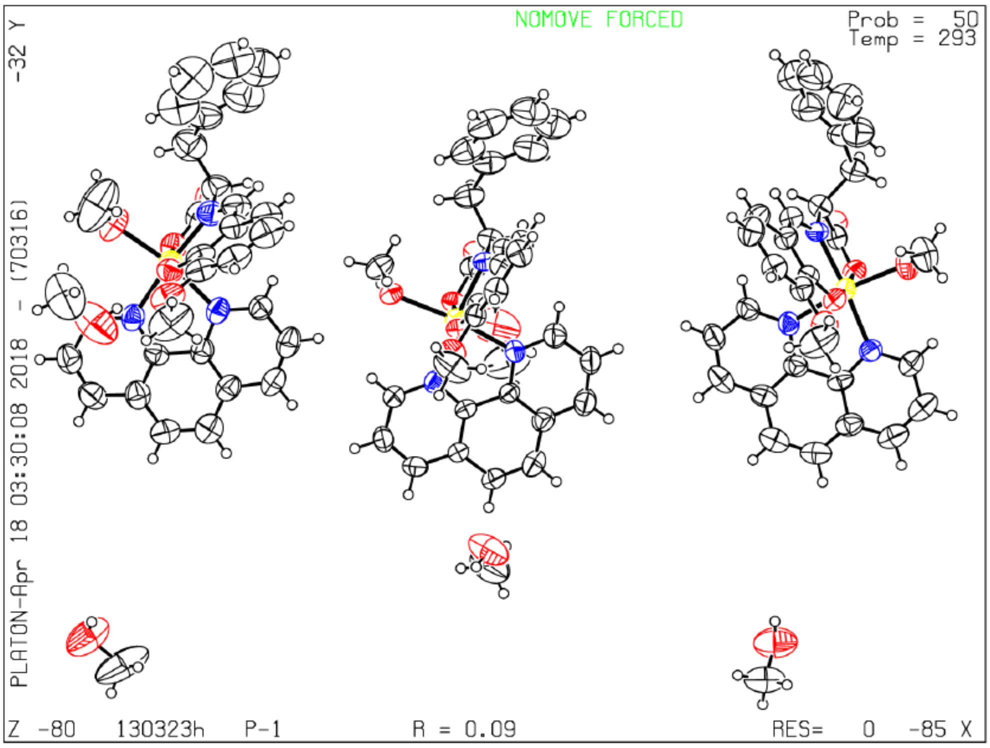

Supplement: Supplementary Materials — Figure S1: mass spectrum of the Schiff base 1. Figure S2: mass spectrum of the Schiff base 2. Figure S3: mass spectrum of the Schiff base 3. Figure S4: 1H NMR spectrum of the Schiff base 1. Figure S5: 1H NMR spectrum of the Schiff base 2. Figure S6: 1H NMR spectrum of the Schiff base 3. CheckCIF/PLATON reports for the crystal structure determinations of complexes 1, 2, and 3. [file 8478152.f1.pdf]
